# Air pollution-related immune gene prognostic signature for hepatocellular carcinoma: network toxicology, machine learning and multi-omics analysis

**DOI:** 10.3389/fimmu.2025.1638445

**Published:** 2025-09-12

**Authors:** Lei Pu, Xiaoyan Zhang, Cheng Pu, Peng Sun

**Affiliations:** ^1^ Key Laboratory of Adolescent Health Assessment and Exercise Intervention of the Ministry of Education, East China Normal University, Shanghai, China; ^2^ College of Martial Arts, Shanghai University of Sport, Shanghai, China

**Keywords:** air pollutants, hepatocellular carcinoma, immune, network toxicology, machine learning, multi-omics analysis

## Abstract

**Background:**

Air pollution may crosstalk with immune system to promote hepatocellular carcinoma (HCC) development, but its precise mechanisms and prognostic significance remain unclear.

**Objective:**

This study aims to construct a prognostic signature for HCC based on air pollutant-related immune genes (APIGs).

**Methods:**

We obtained mRNA-seq and scRNA of HCC from GEO, TCGA and ICGC. AP-related target genes were retrieved from several online databases. APIGs were obtained using WGCNA, differential gene expression analysis and immune infiltration analysis. Molecular subtypes were conducted based on APIG expression to characterize immune features. A total of 101 combinations of 10 machine learning algorithms were used to construct an APIG-based prognostic signature (APIGPS). Furthermore, we performed qRT-PCR, survival analyses, functional enrichment, immune infiltration and single-cell analyses. Subsequently, LASSO, RF, and RFE-SVM were employed to identify diagnostic genes, followed by pan-cancer analysis.

**Results:**

We identified 19 APIGs. HCC samples were divided into 3 subtypes, with C1 exhibiting a pro-tumor immune microenvironment and poorer prognosis. APIGPS constructed by 7 APIGs (CDC25C, MELK, ATG4B, SLC2A1, CDC25B, APEX1, GLS), demonstrated robust predictive ability independent of clinical features. The biological pathway differences between APIGPS-based high- and low-risk groups involved immune responses and cell proliferation and migration. APIGPS genes had stable binding to 7 APs and were mainly expressed in macrophages, with HRG exhibiting higher macrophage abundance. CDC25C was identified as the hub gene after intersecting diagnostic genes and APIGPS genes. CDC25C was associated with survival of 10 cancers, MSI in 10 cancers, TMB in 21 cancers, and immune cell abundance in 13 cancers.

**Conclusions:**

We identified key APIGs and constructed a robust APIG-based prognostic signature for HCC. CDC25C was a key target through which APs impact HCC and multiple other cancers.

## Introduction

The acceleration of global industrialization has exacerbated air pollution, contributing to the onset and progression of various cancers. Air pollutants (APs) and other extrinsic factors account for 70% to 90% of lifetime risk of cancers (1). In 2019, air pollution-related neoplasms caused an estimated 387.45 million death cases globally. And the number of death cases were expected to rise to 559.02 million by 2025 (2). Exposure to pollutants such as PM_2.5_, nitrogen dioxide (NO_2_), SO_2_ and ozone (O_3_) has been strongly associated with the incidence and mortality of multiple cancers, including lung, kidney, colon, bladder cancers, and hepatocellular carcinoma (HCC). However, their exact mechanisms, especially for HCC, remain incompletely understood (3–5).

Air pollution may influence HCC development and prognosis by modulating immune system function. The prognosis for HCC remains poor, with a 5-year survival rate ranging from 13% to 36% from early to advanced stages (6). Among chronic hepatitis B patients treated with nucleotide/nucleoside analogue therapy, air pollution has been associated with an increased risk of developing HCC (7). For every 5.0 μg/m^3^ increase in PM2.5, the hazard ratio for HCC all-cause mortality increased by 1.18 (8). Studies in both animal models and humans suggest that inhalation of APs—such as nitrogen oxides and volatile organic compounds—is associated with hepatic dysfunction. Air pollution may also promote inflammatory responses through immune system crosstalk, enhancing the secretion of pro-inflammatory cytokines such as TNF-α, IL - 6, and IL - 1β (9). However, the precise mechanisms by which air pollution drives HCC via immune modulation remains elusive Based on these studies, we hypothesize that air pollution contributes to HCC progression by modulating tumor immune responses, and that specific air pollutant-related immune genes (APIGs) could serve as valuable diagnostic and prognostic biomarkers. To investigate this, we sought to identify key APIGs and elucidate their biological functions, prognostic significance, and potential therapeutic relevance in HCC.

In this study, we obtained AP-related target genes through network toxicology, and obtained APIGs using weighted gene co-expression network analysis (WGCNA), differential gene expression analysis and immune infiltration analysis. Machine learning algorithms were used to construct a prognostic signature (APIGPS) and validate its predictive performance. Based on the APIGPS, we performed nomogram construction, qRT-PCR, immune infiltration, tumor mutation burden, drug sensitivity, and single-cell analyses. To further explore the potential link between APs and HCC pathogenesis, we further identified diagnostic biomarkers to obtain potential hub genes with both diagnostic and prognostic utility, and performed pan-cancer analysis.

## Methods

### Data collection and processing

**Figure 1:**
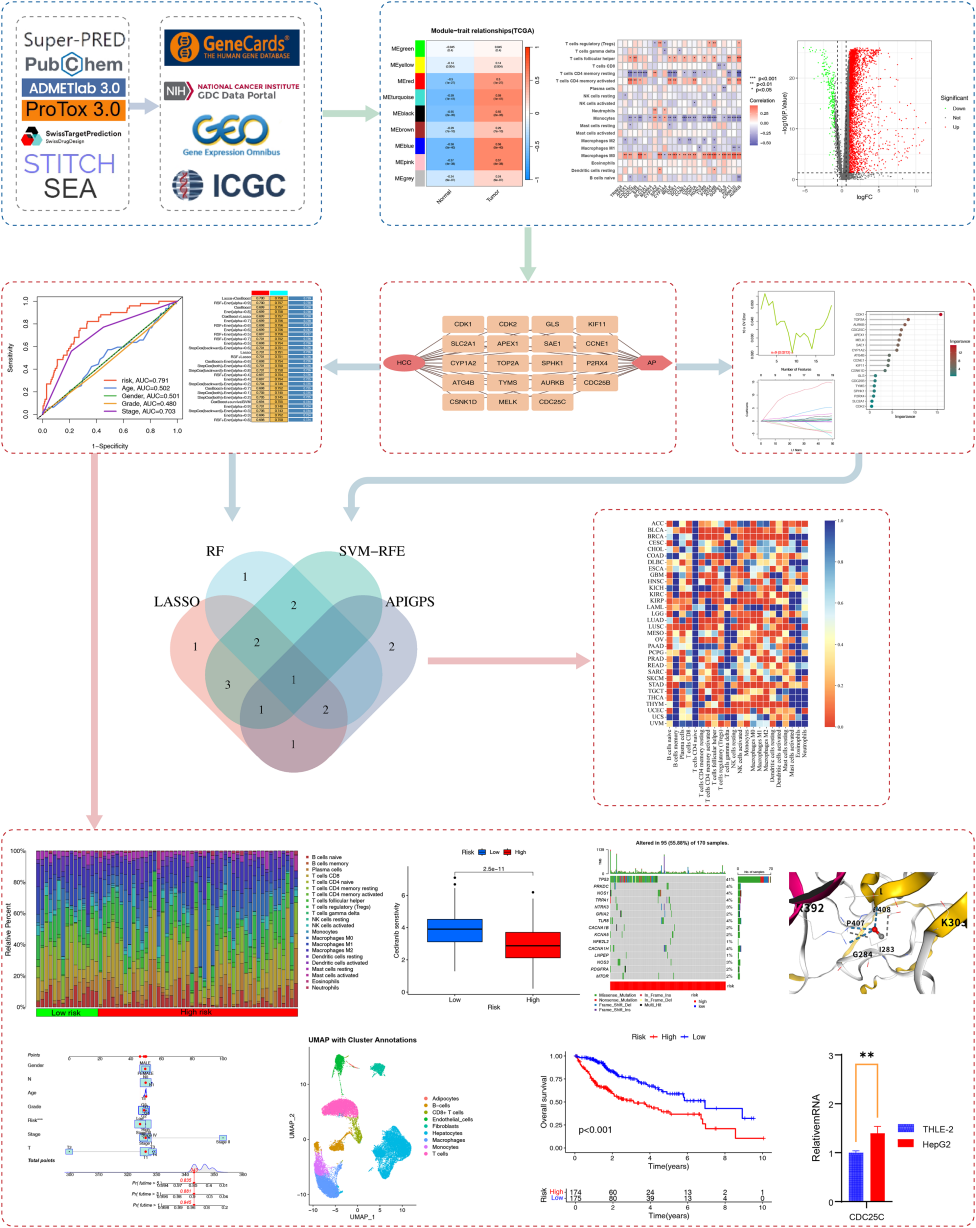
Research flowchart. We utilized multiple databases to obtain AP targets and immune-related targets. Through WGCNA, differential gene expression analysis, and immune infiltration analysis, we identified APIG. Based on these APIG, we constructed the APIGPS and conducted qRT-PCR validation (*=P < 0.05; **=P < 0.01; ***=P < 0.001), survival analysis, immune infiltration assessment, drug sensitivity analysis, tumor mutation burden, nomogram, single-cell analysis, and potential functional enrichment analysis. Subsequently, diagnostic biomarkers were screened based on AP targets and intersected with APIGPS to identify hub gene, followed by pan-cancer analysis.

**Figure 1** presents the research flowchart. We obtained HCC RNA-seq, scRNA-seq, and clinical information from The Cancer Genome Atlas (TCGA; 374 HCC cases; https://portal.gdc.cancer.gov/v1) and the Gene Expression Omnibus (GEO)as the training set. The International Cancer Genome Consortium (ICGC; 233 HCC cases; https://docs.icgc-argo.org/docs/data-access/icgc-25k-data) was used as an external validation set(). We obtained 16206 immune-related genes from GeneCards (www.genecards.org; search term: immune system, score: 1, type: coding; **Supplementary Table S1**).

Chemical structures of 7 APs were retrieved from the PubChem database (https://pubchem.ncbi.nlm.nih.gov/). First, we assessed their carcinogenicity using ADMETLAB 3.0 (https://admetlab3.scbdd.com) and ProTox3 (https://tox.charite.de/protox3). If the result from either database was above 0.5, we considered it indicative of carcinogenicity. Then we obtained human target genes for these compounds using four databases: the SuperPred database (https://prediction.charite.de/; Species: Human; Probability > 50%), Swiss Target Prediction database (http://www.swisstargetprediction.ch/; Species: Human), STITCH database (http://stitch.embl.de/; Organism: Human; score > 0.5), and the SEA database (https://sea.bkslab.org/; Species: Human; P < 0.05).

### Weighted gene co-expression network analysis

Weighted Gene Co-expression Network Analysis (WGCNA) was used to construct gene co-expression networks, identify gene modules with highly coordinated expression patterns, and evaluate module-phenotype relationships. To identify immune genes significantly associated with HCC, we first removed genes with a standard deviation of expression level < 0.5. The optimal soft threshold was determined with a scale-free R^2^ of 0.9. We then converted the adjacency matrix into a topological overlap matrix, and calculated the corresponding dissimilarity (1-TOM). Subsequently, gene modules were identified using dynamic tree cutting and hierarchical clustering (cut height = 0.1, minimum module size = 100).

### Identification of APIGs

We conducted differential gene expression analysis on the 16206 immune-related genes to obtain differentially expressed genes (DEGs) with |log_2_ fold change (FC)| > 1 and FDRq < 0.05. APIGs were obtained by intersecting the identified module genes, DEGs, and AP target genes. To evaluate their correlations with the immune features of HCC, we used the CIBERSORT algorithm (https://CIBERSORT.stanford.edu/) to estimate the infiltration abundance of 22 immune cells in HCC. CIBERSORT uses a deconvolution algorithm to infer cell-type abundances from a complex tissue based on a signature matrix of gene expression profiles. Subsequently, Pearson test was used to assess the correlation between APIGs and the abundance of 22 immune cells. |r| > 0.3 and p < 0.05 denotes a potential correlation.

### Clustering analysis

Based on the expression of APIGs, clustering analysis was conducted to identify molecular subtypes. We used the “ConsensusClusterPlus” R package with Partitioning Around Medoids (PAM) algorithm and Euclidean distance; 80% of the samples were resampled for 10 repetitions. The optimal number of clusters (k) was determined by cumulative distribution function (CDF) plot and average cluster consensus. Principal component analysis (PCA) was conducted to visualize the spatial distribution of subtypes by projecting gene expression data onto principal components (PCs) for dimensionality reduction.

### Functional and pathway enrichment analysis

Gene Ontology (GO) and Kyoto Encyclopedia of Genes and Genomes (KEGG) enrichment analyses were conducted using the “clusterprofiler” R package. We also performed gene set variation analysis (GSVA) to evaluate differentially enriched pathways across samples. Additionally, GeneMANIA (https://genemania.org/) was used for functional network-based enrichment analysis.

### Construction and validation of APIGPS

We used the “sva” R package to remove batch effects in the TCGA and ICGC sets, and then performed univariate COX analysis on the TCGA set to obtain APIGs significantly associated with HCC survival.

Using 10-fold cross-validation, 10 machine learning algorithms—least absolute shrinkage and selection operator (LASSO), Ridge, StepCox, CoxBoost, random survival forest (RSF), generalized boosted regression modeling (GBM), survival support vector machine (survival-SVM), supervised principal components (SuperPC), elastic net (Enet), and partial least squares regression for Cox (plsRcox)—were applied to screen variables to construct 101 APIGPS models. HCC samples with fewer than 20 days of survival and models with fewer than 5 genes were excluded. The optimal model was determined based on the average C-index value. HCC patients were then stratified into high-risk group (HRG) and low-risk group (LRG) according to risk scores calculated through linear combination formula. Subsequently, we performed univariate and multivariate COX analysis, K-M survival analysis, clinical ROC curve, temporal ROC curve, and risk curve using “survival”, “survminer”, and “timeROC” R package to further assess the predicative performance of APIGPS. p < 0.05 and the area under the curve (AUC) > 0.5 were considered significant.

### Correlations with clinical features and nomogram construction

To evaluate the associations between APIGPS and clinical features, we performed chi-square tests followed by K-M survival analysis. Subsequently, we constructed a nomogram using the “rms” R package and evaluated the associations between the nomogram score and HCC survival. C-index value was used to evaluate the consistency of the predicted values and the observed values. Univariate and multivariate COX analyses were used to assess the independent predictive performance of the nomogram, ultimately providing a tool for individualized survival prediction in different HCC patients.

### Immune-related and drug sensitivity analyses

We used CIBERSORT to compare the differences in the infiltration abundance of 22 immune cells between the HRG and LRG. To further validate these differences and evaluate the correlation between APIGPS and immune cell infiltration, we applied 6 additional algorithms, including QUANTISEQ, TIMER, MCPCOUNTER, CIBERSORT−ABS, EPIC, and XCELL. To evaluate immune microenvironment differences between the HRG and LRG, we performed single-sample gene set enrichment analysis (ssGSEA) using the “GSVA” R package, assessing the enrichment scores of 29 immune traits (16 immune cells and 13 immune functions). Based on predefined gene sets, ssGSEA ranks genes according to their expression levels and calculates enrichment scores for individual samples by evaluating the cumulative distribution and weighted enrichment of genes within the set, allowing quantification of the relative activity of specific immune cells in samples.

Next, we utilized the Tumor Immune Dysfunction and Exclusion (TIDE) (http://tide.dfci.harvard.edu/) to assess the immune escape ability of HRG and LRG, where higher scores correlate with poorer response to immunotherapy. Additionally, we obtained the Immuno-Phenoscore (IPS) for HCC patients from the TCIA database, with higher score indicating greater immunotherapy sensitivity. Finally, we leveraged TISIDB (https://cis.hku.hk/TISIDB/index.php) to obtain immune checkpoint genes and assess their differential expression between HRG and LRG, as well as their association with APIGPS in the context of immunotherapy response.

To identify highly sensitive drugs in the HRG and LRG, we used the “oncoppredict” R package to evaluate their sensitivity to 198 FDA-approved drugs. Drug sensitivity was quantified by half maximal inhibitory concentration (IC50), with lower values indicating higher sensitivity.

### Tumor mutational burden analysis

To investigate the link between AP target mutations and APIGPS, we conducted mutation analysis on the 7 AP target genes. Based on the cutoff value, the patients were divided into TMB-high and TMB-low groups, which were then combined with APIGPS for survival analysis. The “maftools” package was used to visualize the top 20 genes with the highest mutation frequencies.

### Single cell analysis

We obtained scRNA-seq data from GEO (GSE210679 and GSE149614) for 11 HCC cases. Data preprocessing was performed using the “Seurat” R package; the percentage of mitochondrial genes was calculated using PercentageFeatureSet. Quality control criteria were: nFeature = 500 - 6000, nCount = 2000 - 40000, and percent.mt < 25. Batch effects were corrected using the “Harmony” package, and PCA were used for dimension reduction, respectively. The top 10 PCs were used for cell clustering via “FindNeighbors” and “FindClusters” functions. Visualization was realized by Uniform Manifold Approximation and Projection (UMAP). Cell type annotation was performed using the “SingleR” package with reference to five human hematopoietic datasets from the “celldex” package (BlueprintEncodeData, MonacoImmuneData, and NovershternHematopoieticData). Furthermore, we performed cell-cell communication analysis using the “CellChat” package, based on ligand-receptor pairs from the CellChat database. Finally, we performed pseudotime trajectory analysis of macrophages using the “monocle” R package, and calculated gene set scores using the AddModuleScore function from the Seurat package.

### Molecular docking

We obtained protein structures of APIGPS genes from the Alphafold (https://alphafold.ebi.ac.uk/) and PDB (https://www.rcsb.org/) databases. Molecular docking analysis was performed using the CB-Dock2 (https://cadd.labshare.cn/), which predicts binding affinity based on structural complementarity. Lower binding energy indicates stronger, more stable binding affinity.

### Identification of hub genes

To further identify key markers with dual diagnostic and prognostic values, we used LASSO, SVM-RFE and RF to screened APIGPS genes based on 10-fold cross-validation. AUC was used to evaluate the diagnostic performance of these genes. LASSO incorporates L1 regularization and the tuning parameter λ (lambda) to control penalty strength. LASSO can shrink the coefficients of redundant features to zero to produce sparse modeling in high-dimensional datasets, which can help identify the most predictive features in disease prediction. SVM-RFE is a feature selection algorithm that combines Support Vector Machine and Recursive Feature Elimination. Its core idea is to use the weight coefficients in the SVM model to rank features and eliminate the least important features iteratively, thus progressively optimizing the feature subset and ultimately the most critical features for improving classification performance. By combining random data sampling and random feature selection, RF constructs multiple decision trees and combines their predictions to select feature genes for disease prediction.

### Pan-cancer analysis of the hub gene

Given the widespread impact of APs on the survival of various cancers, we performed pan-cancer analysis using mRNA-seq data of 33 cancers retrieved from the UCSC database (https://xena.ucsc.edu/). We examined the expression level of the hub gene in 33 cancers and the association with immune infiltration, pan-cancer survival, tumor mutational burden (TMB), and microsatellite instability (MSI).

### qRT-PCR and immunohistochemistry

The cell lines (THLE - 2, HepG2) were purchased from the Institute of Cell Research, Chinese Academy of Sciences. Total RNA was isolated using Trizol reagent following the manufacturer’s instructions (Invitrogen, 1596 - 026). cDNA was synthesized using a reverse transcription kit (Fermentas, #K1622). Quantitative real-time PCR (qRT-PCR) was conducted with the SYBR Green kit (Thermo, #K0223). GAPDH served as the internal control for normalization. The primer sequences are provided in **Supplementary Table S6**.

Immunohistochemical (IHC) results for normal people and HCC patients were obtained from the Human Protein Atlas (HPA) database (https://www.proteinatlas.org/).

### Statistical analysis

All statistical analyses were performed using R software (Version 4.3.2; the R Foundation, St. Louis, MO, USA). Chi-square test was used for categorical data; t-test or Wilcox test was used for continuous data. Cytoscape (v3.8.2) was used to visualize the interaction network. HR > 1 was considered a risk factor; HR < 1 indicated a protective factor. Unless otherwise specified, p < 0.05 with a confidence interval of 95% was considered significant.

## Results

### Toxicity and target genes of air pollutants

The 7 Aps analyzed exhibited high carcinogenicity in both toxicity prediction platforms. Target genes were identified from four databases as follows: 82 for benzene, 70 for carbon monoxide (CO), 92 for nitric oxide (NO), 87 for nitrogen dioxide (NO_2_), 88 for ozone (O_3_), 92 for sulfur dioxide (SO_2_), and 102 for toluene. After intersection, a total of 257 AP-related target genes were identified (**Supplementary Tables S1**, **S2**).

### Identification of APIGs associated with HCC

**Figure 2:**
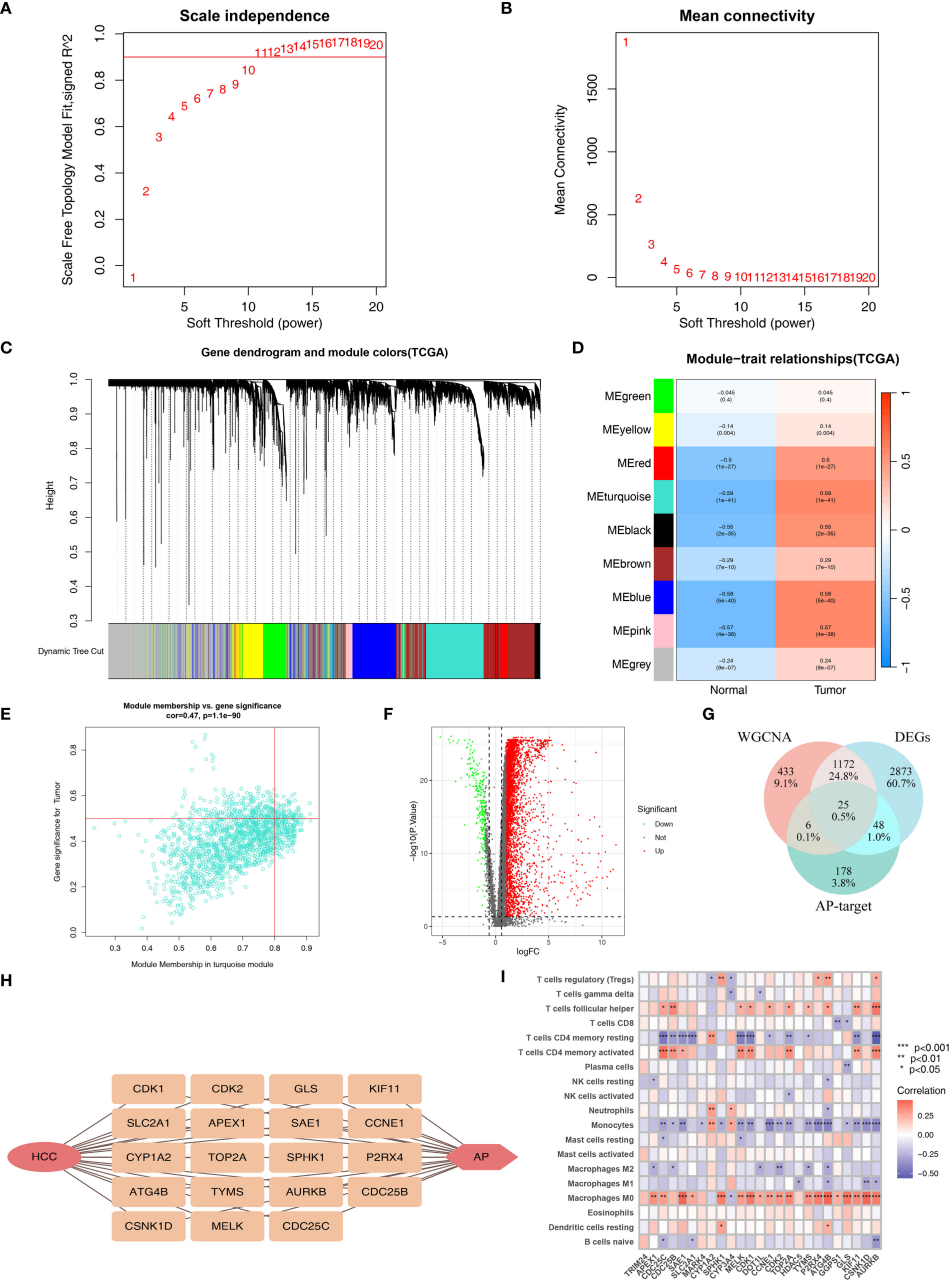
Identification of APIG. **(A)** The selection of soft threshold β. **(B)** Mean connectivity for HCC. **(C)** Cluster dendrogram of WGCNA analysis. **(D)** Module-trait heatmap indicating the correlation between modules and HCC. **(E)** The correlation between module membership and gene significance in the turquoise module for HCC. **(F)** Volcano plot of DEGs. **(G)** Venn diagram of DEGs, module genes, and AP-target. **(H)** Correlation plot of APIG. **(I)** Correlation analysis between genes and immune infiltration (*=P < 0.05; **=P < 0.01; ***=P < 0.001).

We performed WGCNA using the TCGA set to identify immune genes most associated with HCC. Based on the optimal power value of 11 (**Figures 2A, B**), we identified 9 co-expression modules (**Figure 2C**). Among these modules, the turquoise module (containing 1636 genes) had the highest correlation with HCC (r=0.59, p=1e-41; **Figure 2D**). And the gene significance and module membership for the turquoise module showed a significant correlation (r=0.47, p=1.1e-90; **Supplementary Table S3**; **Figure 2E**).

Subsequently, differential gene expression analysis of 16206 immune genes identified 4118 DEGs (**Supplementary Table S4**; **Figure 2F**). Intersecting these DEGs with 1636 genes in the turquoise module yielded1197 immune genes associated with HCC.

These 1197 immune genes were further intersected with the 257 AP target genes, resulting in 25 genes (**Figures 2G, H**). We then assessed their correlation with immune cell infiltration and finally obtained 19 APIGs (**Supplementary Table S5**; **Figure 2I**).

### Molecular subtyping based on APIGs

**Figure 3:**
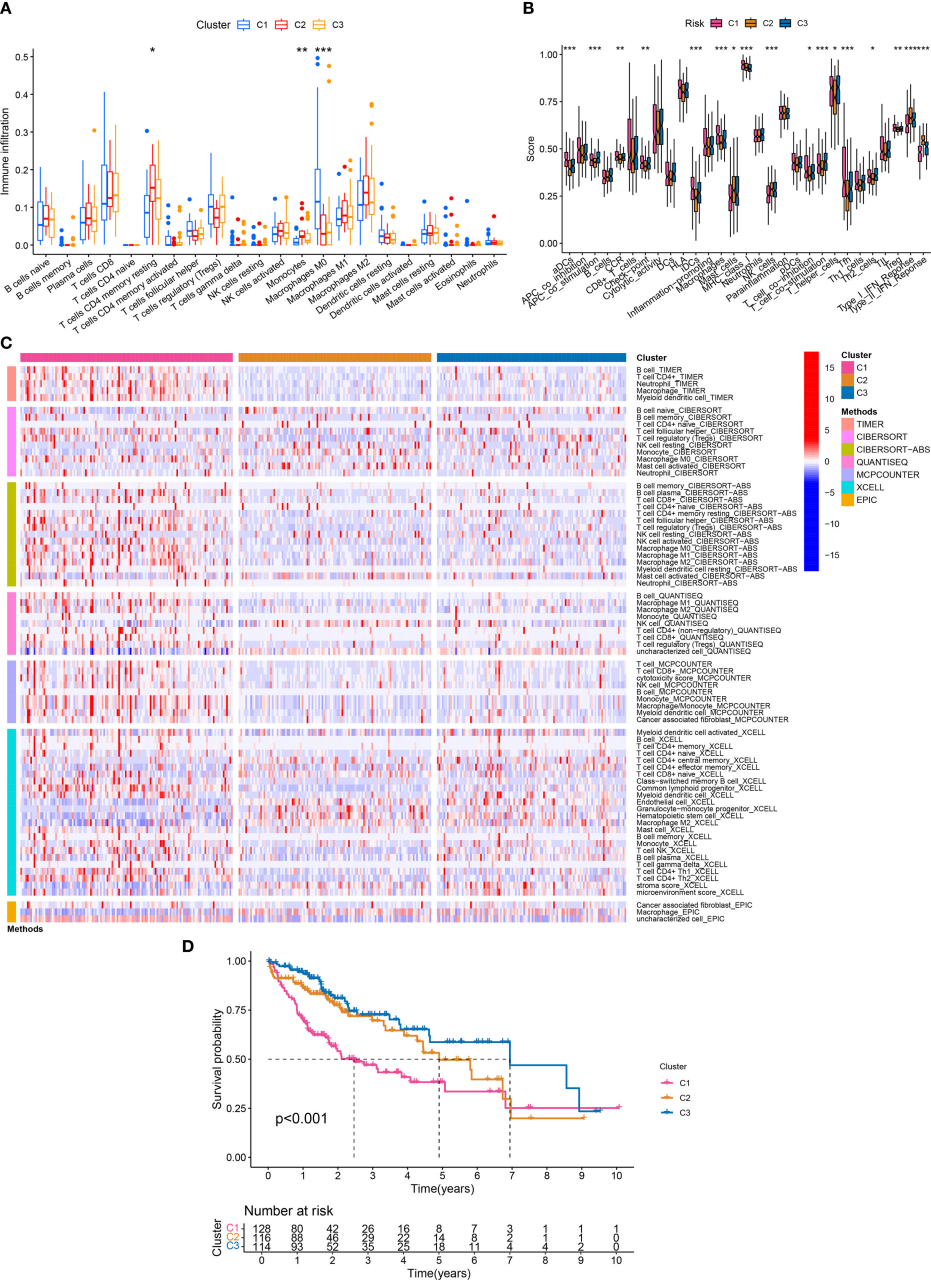
Molecular subtyping and immune traits. **(A)** Differences in the infiltration abundance of 22 immune cells between the three subtypes (*P < 0.05; **P < 0.01; ***P < 0.001). **(B)** Differences in the infiltration abundance of 29 immune traits between the three subtypes (*P < 0.05; **P < 0.01; ***P < 0.001). **(C)** 7 immune infiltration algorithms for the three subtypes. **(D)** Survival analysis of the three subtypes.

Based on the expression of APIGs and CDF curve, HCC samples were divided into 3 subtypes (**Supplementary Figures S1A, B**). PCA showed distinct separation among the subtypes (**Supplementary Figure S1C**). Among them, C1 subtype exhibited a tumor-promoting immune phenotype, characterized by significantly higher macrophage (Mφ) abundance as confirmed by both ssGSEA and CIBERSORT analyses (**Figures 3A, B**). In contrast, C2 and C3 subtypes exhibited features of an immunosuppressive microenvironment, such as higher abundance of resting memory CD4^+^ T cells and monocytes, yet displayed a more favorable immune microenvironment than C1. Consistently, all 7 immune infiltration algorithms confirmed the significant differences in Mφ abundance between C1 and C2/C3 subtypes (**Figure 3C**). As expected, the K-M curve indicated that the C1 subtype had significantly lower overall survival compared to C2 and C3 subtypes (**Figure 3D**). These findings suggest that AP-related immune alterations may drive distinct immune subtypes in HCC.

### Construction of APIGPS through machine learning

**Figure 4:**
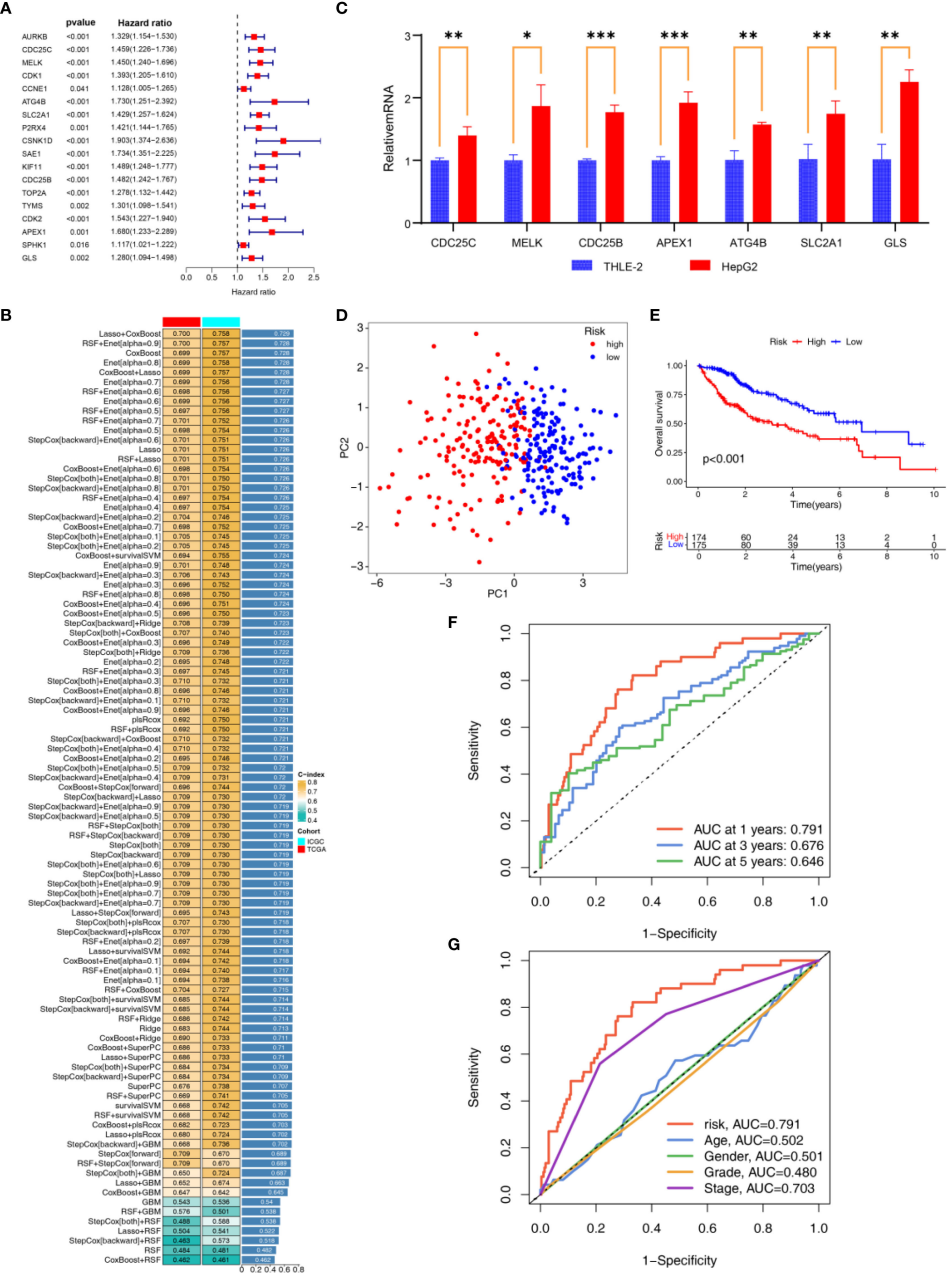
Construction and Validation of APIGPS. **(A)** Univariate COX analyses associated with survival. **(B)** Construction of APIGPS using integrated machine learning. **(C)** qRT-PCR of the signature genes (*=P < 0.05; **=P < 0.01; ***=P < 0.001). **(D)** PCA based on EIGPS. **(E)** K-M survival curves based on APIGPS. **(F)** ROC curves predicting 1-, 3-, and 5-year survival. **(G)** ROC curves of risk scores and clinical features.

Univariate COX analysis identified 18 APIGs significantly associated with HCC prognosis (**Figure 4A**; **Supplementary Table S6**). Subsequently, based on 10-fold cross validation, 101 combinations of 10 machine learning algorithms were employed to construct APIGPS models. Among them, APIGPS constructed by Lasso+CoxBoost had the highest average C-index (0.729; **Figure 4B**), and incorporated 7 APIGs (CDC25C, MELK, ATG4B, SLC2A1, CDC25B, APEX1, GLS). K-M survival analysis indicated that low expression of these genes was associated with better HCC survival (**Supplementary Figures S2A-G**). Additionally, qRT-PCR confirmed their significant upregulation in HCC samples (**Figure 4C**; **Supplementary Table S7**), which was further supported by IHC results (**Supplementary Figures S3A-G**).

### Evaluation and validation of APIGPS

To assess the performance of the constructed APIGPS, we calculated risk scores for patients and divided them into HRG and LRG. PCA demonstrated clear separation between HRG and LRG (**Figure 4D**). Notably, increasing APIGPS risk scores correlated with progressively higher mortality rates (**Supplementary Figures S4A, B**). K-M curve showed that HRG had significantly worse survival than LRG (**Figure 4E**). Time-dependent ROC curves suggested that the APIGPS had good predictive accuracy for 1-year (AUC = 0.791), 3-year (AUC = 0.676), and 5-year (AUC = 0.646) survival (**Figure 4F**). Importantly, APIGPS outperformed other clinical features in prognostic accuracy (**Figure 4G**). Univariate (HR = 3.958, 95%CI: 2.527 - 6.193, P<0.001; **Supplementary Figure S4C**) and multivariate COX regression analyses (HR = 3.113, 95%CI:1.904-5.091, P<0.001; **Supplementary Figure S4D**) confirmed the independent prognostic value of APIGPS. Furthermore, APIGPS exhibited potential associations with molecular subtyping (**Supplementary Figure S5**).

We further validated the robustness and generalizability of APIGPS in the external validation set. The results of the ROC curve (**Supplementary Figures S6A, B**), K-M analysis (**Supplementary Figure S6H**), risk curve (**Supplementary Figure S6C**), PCA (**Supplementary Figure S6G**), scatter plot (**Supplementary Figure S6F**), univariate (**Supplementary Figure S6E**) and multivariate (**Supplementary Figure S6B**) COX regression analyses, and signature genes expression (**Supplementary Figure S6I**) in the validation set were all consistent with the training set.

### Clinical correlations and the nomogram based on APIGPS

We evaluated the correlations between APIGPS and clinical features and observed significant differences in T-stage, Stage, and Grade between HRG and LRG (**Supplementary Figure S7A**). K-M analysis revealed that the prognostic value of APIGPS was independent of clinical features: patients in the LRG consistently had better survival across early and late stage (**Supplementary Figures S7B-D**).

To enable personalized prognostic prediction for HCC patients, we constructed a nomogram based on APIGPS (**Supplementary Figure S8A**). The nomogram accurately predicted 1-, 3-, and 5-year survival (C-index = 0.736, 95% CI: 0.685 - 0.787; **Supplementary Figure S8B**). Mortality gradually increased with higher nomogram scores (**Supplementary Figure S8C**). Both univariate (HR = 2.061, 95%CI:1.712-2.48, P<0.001; **Supplementary Figure S8D**) and multivariate (HR = 1.9, 95%CI:1.428-2.53, P<0.001; **Supplementary Figure S8E**) COX regression analyses confirmed the nomogram’s independent prognostic utility (AUC = 0.832; **Supplementary Figure S8F**).

### Immune infiltration analysis based on APIGPS

**Figure 5:**
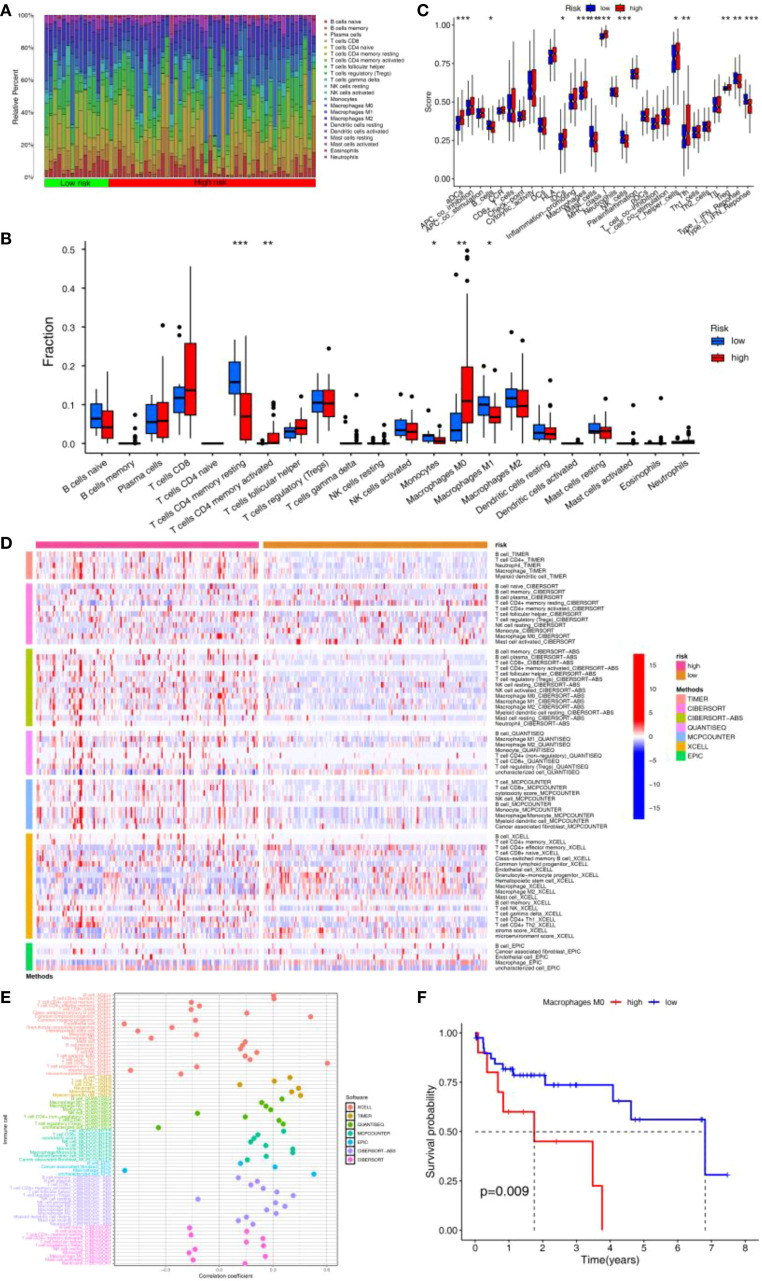
Immune infiltration analysis of APIGPS. **(A)** Heatmap of immune infiltration in HRG and LRG. **(B)** Differences in 22 immune cells in HRG and LRG (*P < 0.05; **P < 0.01; ***P < 0.001). **(C)** Differences in 16 immune cells and 13 immune functions in HRG and LRG (*P < 0.05; **P < 0.01; ***P < 0.001). **(D)** Heatmap of the differences in the immune infiltration abundance between HRG and LRG based on 7 immune infiltration algorithms. **(E)** Correlation between risk scores and immune infiltration abundance based on 7 immune infiltration algorithms. **(F)** Survival analysis of macrophages.

CIBERSORT analysis indicated that the abundance of Mφ and activated memory CD4^+^ T cells were significantly higher in HRG than in LRG, while the abundance of resting memory CD4^+^ T cells, monocytes, and M1-like macrophage (M1)were significantly higher in LRG than in HRG (**Figures 5A, B**). Consistently, ssGSEA showed higher activity or abundance of immature dendritic cells (iDCs), Mφ, MHC class I, CD4^+^ T helper cells, and tumor-infiltrating lymphocytes (TILs)in the HRG than in LRG (**Figure 5C**). Results from 7 immune infiltration algorithms further supported an elevated abundance of multiple tumor-promoting immune features in the HRG (**Figure 5D**). For instance, Mφ abundance was significantly elevated across multiple algorithms, and was significantly positively correlated with APIGPS (**Figure 5E**). Furthermore, all of these algorithms showed that Mφ abundance was elevated in HRG. Survival analysis demonstrated that higher Mφ abundance was associated with worse survival (**Figure 5F**).

### Single cell analysis based on APIGPS

**Figure 6:**
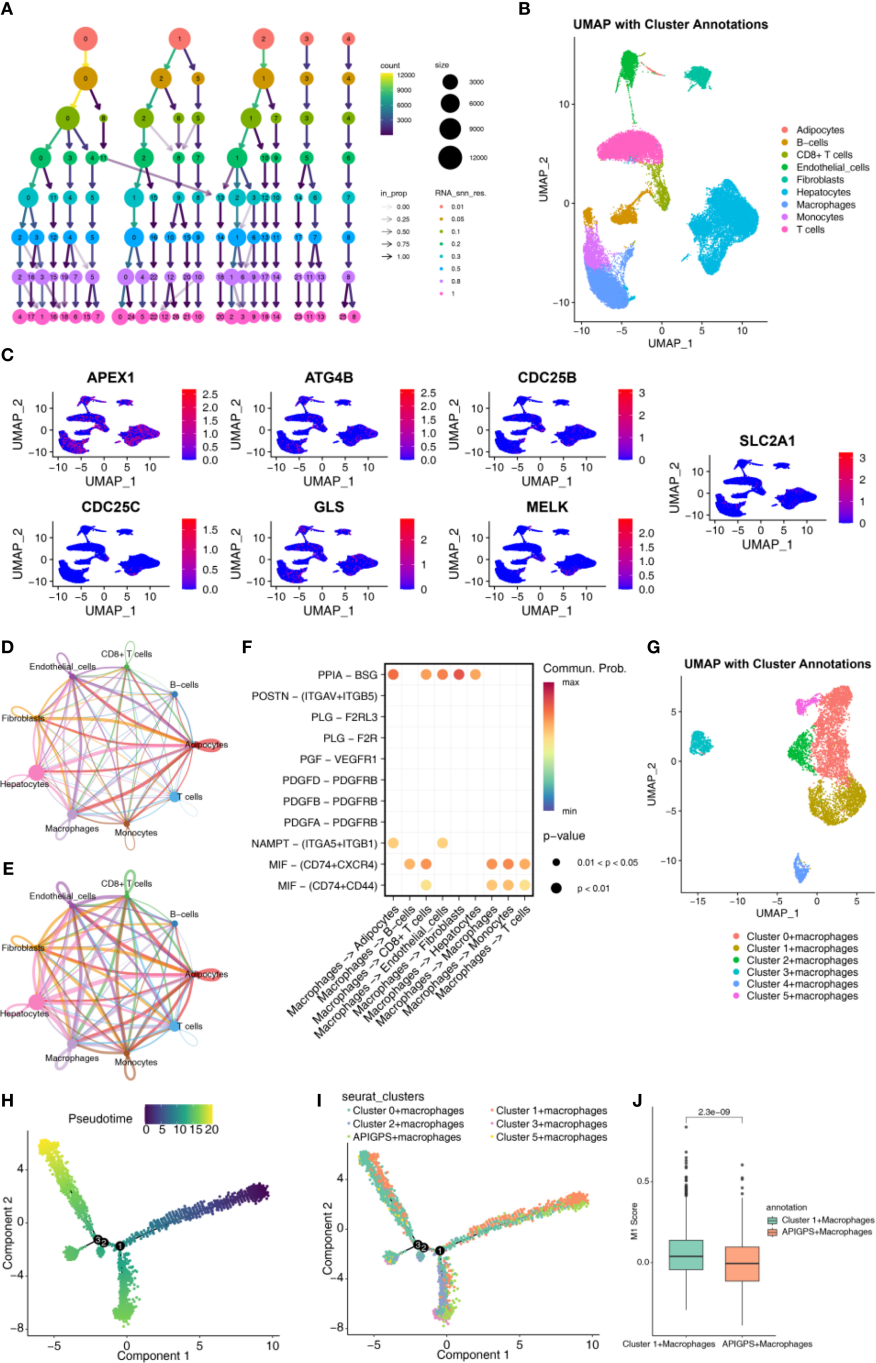
Single cell analysis. **(A)** Annotated cell types. **(B)** Selection of clustering resolutions. **(C)** APIGPS genes expression in cells. **(D)** Circle plot of interaction number in cell-cell communication. **(E)** Circle plot of interaction strength in cell-cell communication. **(F)** Receptor-ligand for cell communication. **(G)** Annotation of macrophages. **(H)** Macrophage pseudotime trajectory. **(I)** The pseudotime trajectory of macrophage classification. **(J)** The score of M1.

After quality control, we obtained 33694 genes and 43918 cells; after outlier removal, we retained 25190 genes and 34202 cells for normalization and dimensionality reduction (**Supplementary Figure S9A**). The top 10 PCs were selected according to the elbow plot (**Supplementary Figure S9B**). We identified 18 clusters with 0.5 as the best resolution (**Figure 6A**; **Supplementary Figures S9C, D**). Cell type annotation revealed cell types, including adipocytes, B cells, CD8^+^ T cells, endothelial cells, fibroblasts, hepatocytes, Mφ, monocytes and T cells (**Figure 6B**). APIGPS genes were mainly expressed in Mφ (**Figure 6C**). Cell–cell communication analysis showed that interactions between Mφ and endothelial cells exhibited the highest interaction number and strength (**Figures 6D, E**; **Supplementary Tables S8** and **S9**), with PPIA-BSG ligand-receptor pairs playing a mediating role (**Figure 6F**). Subsequently, we extracted macrophage subsets and identified distinct macrophage clusters (**Figure 6G**). Using the AddModuleScore function, we calculated APIGPS scores and found that cluster 4 exhibited the highest scores (**Supplementary Figure S9E**). Pseudotime trajectory analysis revealed three distinct differentiation paths (**Figure 6H**), with APIGPS^+^ macrophages located at the terminal end of the first trajectory (**Figure 6I**). Notably, along this trajectory, the APIGPS^+^ cells showed a phenotypic shift from high to low M1 polarization scores (**Figure 6J**), suggesting a potential inhibitory effect of APIGPS on M1 polarization.

### Macrophage-related immune pathway enrichment analysis based on APIGPS

To delineate differences in Mφ-related pathways between HRG and LRG, we performed GO and KEGG analyses. The most significant differences in immunological functions and pathways were leukocyte mediated immunity and chemokine signaling pathway, respectively (**Supplementary Figures S10A, B**; **Supplementary Tables S10**, **S11**). GSVA further confirmed dysregulated Mφ-related pathways in HRG, such as complement and coagulation cascades, and Fc gamma receptor (FcγR) mediated phagocytosis (**Supplementary Figure S10C**; **Supplementary Table S12**).

### Immunotherapy-related and drug sensitivity analyses based on APIGPS

**Figure 7:**
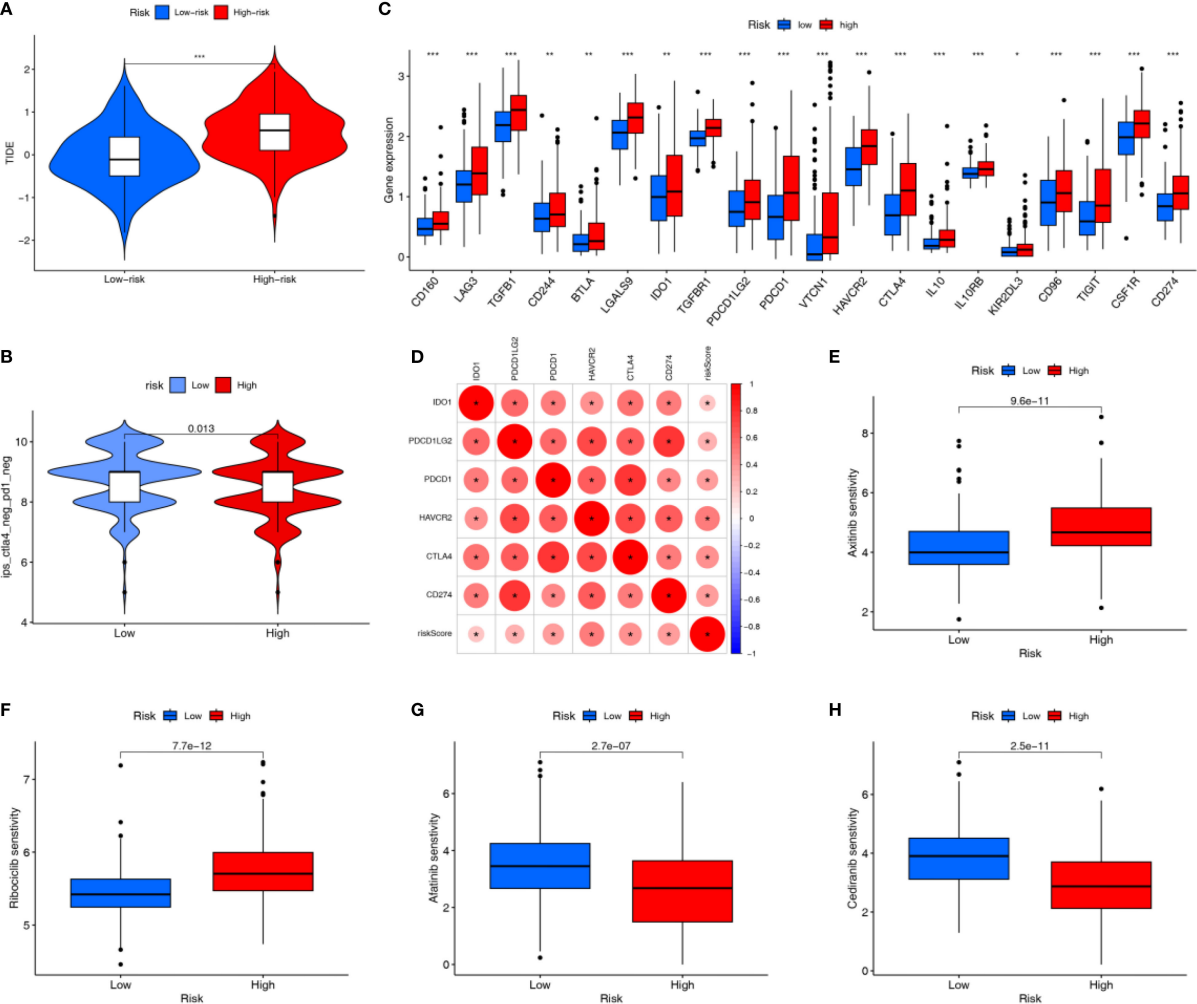
Immunotherapy related analysis. **(A)** TIDE scores (***P < 0.001). **(B)** IPS scores. **(C)** Differential expression of immune checkpoint genes (*P < 0.05; **P < 0.01; ***P < 0.001). **(D)** Correlation of risk scores with clinically common immune checkpoints. **(E, F)** Drugs that are more sensitive in the LRG. **(G, H)** Drugs that are more sensitive in the HRG.

Immunotherapy represents a promising strategy for the treatment of HCC. The HRG had a significantly higher TIDE score than the LRG, suggesting its greater immune escape ability and poorer predicted response to immunotherapy (**Figure 7A**). The LRG had a higher IPS for CTLA4-/PD - 1- treatment, suggesting its better response to PD - 1 inhibitor and CTLA4 inhibitor therapy (**Figure 7B**). Immune checkpoint-related genes are closely associated with the efficacy of immunotherapy, and were more highly expressed in the HRG (**Figure 7C**). The expression of immune checkpoint-related genes can reflect T-cell activation or exhaustion, and higher expression can indicate either immune activation or immune suppression. Given our immune infiltration findings, this likely reflects an immune-suppressive phenotype in the HRG. Moreover, APIGPS risk scores were positively correlated with the expression of core clinical immunotherapy targets, implying that HCC patients with higher risk scores may derive less benefit from immunotherapy (**Figure 7D**). Drug sensitivity analysis indicated that the LRG was more sensitive to Axitinib and Ribociclib (**Figures 7E, F**), whereas the HRG showed higher sensitivity to Afatinib and Cediranib (**Figures 7G, H**).

### Tumor mutational burden analysis based on APIGPS

After dividing HCC patients into TMB-high and TMB-low groups, K-M curves showed that TMB-high group had significantly lower survival rate than TMB-low group (**Supplementary Figure S11A**). Mutation rates differed markedly between risk groups, with 55.8% in HRG and 32.35% in LRG (**Supplementary Figures S11B, C**). Among the 257 AP-related target genes, TP53 was the most frequently mutated gene in both HRG and LRG. When combining TMB status with APIGPS risk, the TMB-high + HRG group had the worst survival (**Supplementary Figure S11D**).

### Identification of a hub gene with dual diagnostic and prognostic value

**Figure 8:**
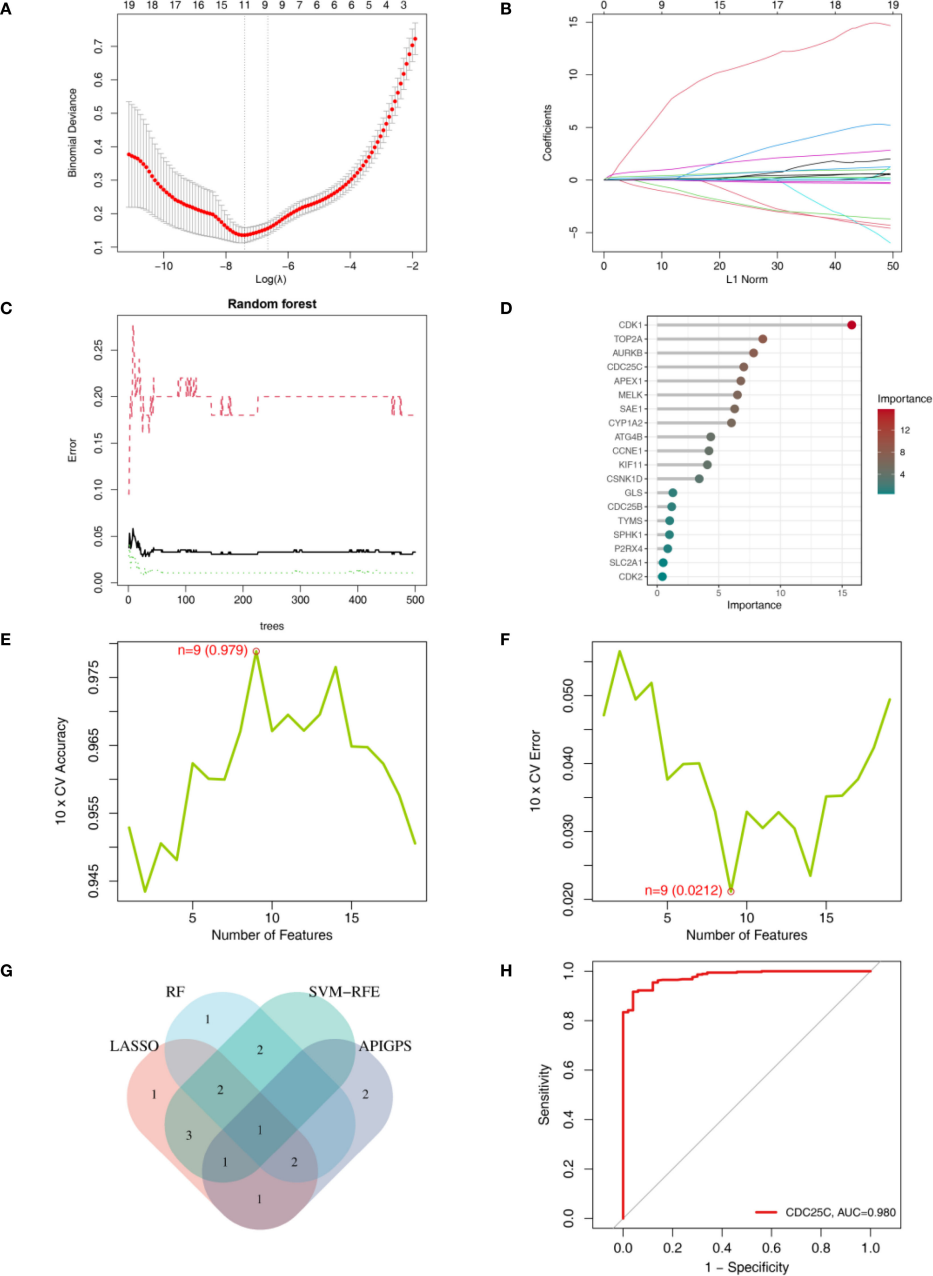
Identification of hub gene using machine learning. **(A)** LASSO lambda. **(B)** LASSO coefficient profiles. **(C)** RF error rate. **(D)** RF importance ranking of AP-related targets. **(E)** SVM-RFE accuracy of AP-related targets. **(F)** SVM-RFE error of AP-related targets. **(F)** RF importance ranking of AP-related targets. **(G)** Venn diagram of genes identified by 3 machine learning methods. **(H)** ROC curve of CDC25C.

We employed LASSO, SVM-RFE, and RF to screen key genes from 19 APIGs. LASSO selected 11 genes based on the minimum λ (**Figures 8A, B**); RF identified 8 genes with an importance score > 5 (**Figures 8C, D**); SVM-RFE identified 9 genes according to the minimum error (**Figures 8E, F**; **Supplementary Table S13**). After intersecting these genes with APIGPS genes (**Figure 8G**), CDC25C was identified as the hub gene with good diagnostic value (AUC = 0.98; **Figure 8H**).

### Pan-cancer analysis of the hub gene

To explore broader oncogenic relevance, we conducted a pan-cancer assessment of CDC25C. CDC25C was highly expressed in all cancers and was involved in cell cycle-related functions (**Supplementary Figures S12A, B**). In addition, CDC25C was significantly associated with immune cell abundance in 13 cancers (**Supplementary Figure S12C**; **Supplementary Table S14**), TMB in 21 cancers (**Supplementary Figure S12D**; **Supplementary Table S15**), and microsatellite instability in 10 cancers (**Supplementary Figure S12E**; **Supplementary Table S16**). High CDC25C expression was associated with low survival in 9 cancers besides HCC (**Supplementary Figures S13A-I**).

### Molecular docking analysis based on APIGPS

**Figure 9:**
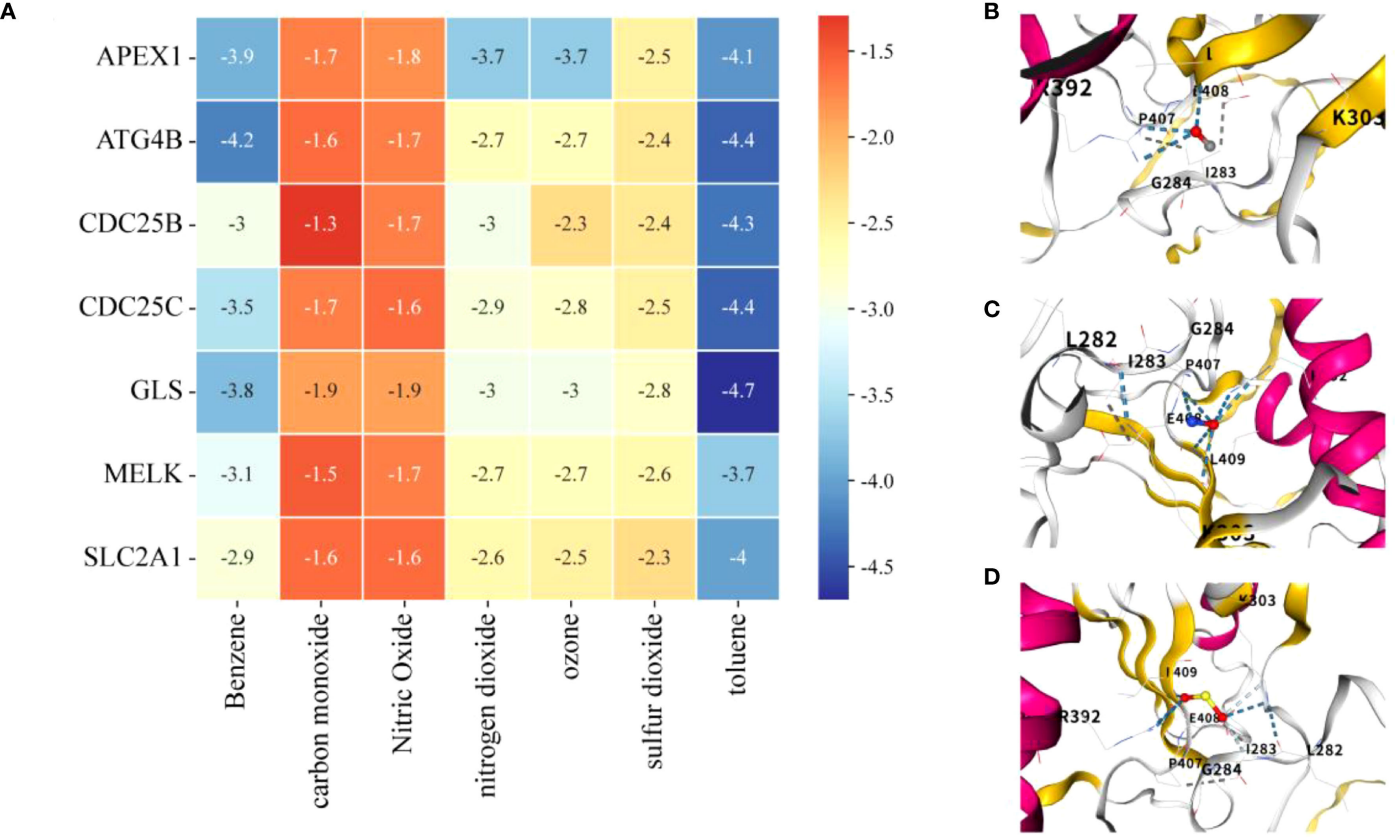
Molecular docking. **(A)** Heatmap of binding energies of molecular docking analysis (lower energy indicates stronger binding affinity). **(B)** CDC25C and carbon monoxide. **(C)** CDC25C and nitric oxide. **(D)** CDC25C and sulfur dioxide.

Molecular docking analysis suggested that all AP–APIGPS interactions had binding energies between -1 to -5, indicating good binding affinity between 7 APs and 7 APIGPS genes (**Figure 9A**; **Supplementary Table S17**). Among them (Top 3), CDC25C formed more unstable bindings with carbon monoxide (**Figure 9B**), nitric oxide (**Figure 9C**), and sulfur dioxide (**Figure 9D**). These findings further supported the hypothesis that APs may crosstalk with APIGPS expression, thereby influencing the progression of HCC.

## Discussion

The rising mortality and morbidity of air pollution-induced HCC underscore an urgent public health concern. Recent epidemiological evidence suggests that air pollution may interact with the immune system to promote HCC progression; however, the exact mechanisms remain unclear. In this study, we intersected AP-related target genes (derived from network toxicology) with HCC-related immune genes (identified via WGCNA and differential gene expression analysis), followed by immune infiltration analysis, and ultimately identified 19 APIGs for molecular subtyping. Subsequently, we constructed an APIGPS containing 7 APIGs, which demonstrated robust predictive performance. Further analysis revealed that Mφ were a major immune cell phenotype distinguishing HRG and LRG, with HRG exhibiting poorer response to immunotherapy. Afatinib and Cediranib were identified as potential sensitive drugs for HRG patients. Single-cell RNA sequencing showed that APIGPS genes were predominantly expressed in Mφ. TP53 was identified as the most frequently mutated gene. Using 3 machine learning methods, we identified diagnostic genes and intersected them with APIGPS, identifying CDC25C as the hub gene. Finally, molecular docking analysis confirmed strong binding affinity between 7 APs and 7 APIGPS genes, which further supported the hypothesis that AP may drive the occurrence and development of HCC through molecular interactions with APIGPS.

To the best of our knowledge, this study is the first to identify APIGs highly correlated with HCC and constructed a clinically applicable APIGPS. HCC patients were classified into three subtypes based on the 19 APIGs, with C1 subtype exhibiting tumor-promoting immune phenotype, particularly elevated Mφ abundance, and the poorest prognosis. These findings suggest that AP exposure may induce distinct HCC subtypes by modulating the immune microenvironment, thus complicating personalized treatment strategies. APIGPS demonstrated good predictive performance; the HRG had poorer prognosis and overlapped mostly with C1 subtype, suggesting that APIGPS may reflect subtype-specific molecular and immunological features. Multivariate COX analysis and ROC curves validated the independence and robustness of APIGPS in both the training and external validation set. To aid clinical application, we constructed a nomogram for better individualized prognosis prediction. Furthermore, CDC25C showed good diagnostic value and may be involved in the progression of multiple cancers, making it an important pan-cancer target for APs.

CDC25C is a cell cycle–regulating phosphatase that primarily promotes G2/M transition by activating the CDK1/cyclin B complex (10). Although not traditionally considered an immune regulator per se, CDC25C is involved in immune modulation by inducing genomic instability. It is highly expressed in multiple cancers and correlates with TMB, microsatellite instability, immune infiltration, and low survival in at least 10 cancers. Overexpression of CDC25C can counteract the suppression by the ATM/ATR-CHK1/2 pathway, leading to sustained CDK1 activation. This enables DNA-damaged cells to bypass the checkpoints and enter M phase, promoting genomic instability and mutations in tumor suppressor genes; it also disrupts the DNA mismatch repair system, ultimately accelerating tumor cell proliferation (10, 11). Additionally, such instability produces cytosolic DNA fragments, which are recognized by cGAS and catalyze the synthesis of the second messenger cGAMP. cGAMP binds to and activates STING protein on the endoplasmic reticulum membrane, inducing its conformational change and recruiting TBK1 kinase. Activated TBK1 phosphorylates IRF3, promoting its nuclear translocation and initiating IFN-α/β transcription, which rapidly recruits CD8^+^ T cells and NK cells to elicit anti-tumor effects (12, 13). However, chronic overexpression of CDC25C upregulates PD-L1 in tumor cells through the STAT3/IRF1 pathway, and promotes the recruitment of Tregs, MDSCs and M2-like macrophages (M2) via chemokines such as CXCL10. These immunosuppressive cells directly inhibit T cell activity by secreting IL - 10, Arg1, and TGF-β, and enhance angiogenesis, tumor invasion, and immune escape by secreting factors like VEGF and MMP9 (14–17). Furthermore, persistent activation of the ATM/ATR signaling pathway caused by DNA damage repair defects can also upregulate the expression of immune checkpoints such as PD-L1 and VISTA via the NF-κB or HIF - 1α pathways (17). CDC25C overexpression has been associated with shorter progression-free survival in LUAD patients treated with nivolumab (18). Targeted inhibition of CDC25C induces immunogenic cell death, leading to the release of damage-associated molecular patterns (19, 20). Extracellular ATP activates the P2X7 receptor on Mφ surface, inducing NLRP3 inflammasome-dependent IL - 1β release and upregulating the expression of MHC-II molecules and costimulatory molecules CD80/CD86, thereby promoting the cross-presentation of tumor antigens to CD4^+^ T cells (21, 22). HMGB1, on the other hand, activates the NF-κB pathway in Mφ via TLR4/RAGE signaling, promoting the secretion of chemokines such as CXCL9/CXCL10 and recruitment of effector T cells into the tumor microenvironment, while inhibiting Mφ polarization towards the M2 phenotype (20, 23).

The other 6 APIGs in the APIGPS also act as poor prognostic factors. ATG4B, a member of C54 peptidases, primarily cleaves the C-terminus of microtubule-associated protein 1 light chain 3 to promote the extension and closure of autophagosome membrane (24). Targeted inhibition of ATG4B can block ATG4B-mediated autophagic degradation of TBK1 (a crucial kinase for antiviral immunity), enhancing antiviral immune responses and CD8^+^ T cell infiltration, thereby delaying HCC progression (25, 26). Additionally, ATG4B closely interacts with SLC2A1, promoting the Warburg effect in tumor and increasing L-lactate production and glucose uptake (27). SLC2A1, in turn, facilitates glucose transport to maintain the Warburg effect, and promotes Mφ polarization toward the M2 by enhancing efferocytosis and inflammatory factors secretion such as CD206 and IL - 10 (28). In liver metastatic lesions, SLC2A1 fosters an immunosuppressive microenvironment by increasing the proportion of Mφ and their inhibitory interactions with T cells (29). Furthermore, SLC2A1 interacts with GLS, mediating the transport of glutamine into the cell. GLS catalyzes the hydrolysis of glutamine to glutamate, the rate-limiting step in glutamine metabolism, to generate α-ketoglutarate for the TCA cycle (30). GLS-driven glutaminolysis increases production of α-ketoglutarate and reactive oxygen species, which activate the Wnt/β-catenin signaling pathway and maintain the stemness and survival of cancer stem-like cells (31). This process contributes to immune suppression by reducing T cell infiltration and promoting the expression of immune checkpoint molecules (32). Moreover, excessive glutamine metabolism mediated by GLS1 drives the polarization of Mφ toward the M2 immunosuppressive phenotype and inhibits Th1 and CD8^+^ T cell differentiation (33). APEX1 is a DNA repair enzyme with apurinic/apyrimidinic activity. Its redox domain regulates the activity of several transcription factors such as NF-κB, AP - 1, and STAT3, enhancing cytokine and chemokine secretion such as TNFα, IL - 6, and IL - 8, ultimately creating a pro-inflammatory and immunosuppressive microenvironment (34, 35). Targeted inhibition of APEX1 or its redox function can enhance the IFNγ-producing Th1 response and suppress HCC cell migration and proliferation (36, 37). MELK, a serine/threonine protein kinase, is a cell-cycle modulator essential for mitotic progression (38). MELK promotes HCC cell migration by upregulating MMP7 expression and regulates G2/M phase progression via PLK1-CDC25-CDK signaling, thereby inhibiting apoptosis (38). This process may further suppress CD8^+^ T cell infiltration by activating downstream cascades such as CDK4/6 activation, facilitating tumor immune escape (39). Knockdown of MELK inhibits cell viability by inducing apoptosis and mitosis in HCC cells, promotes M1polarization, hinders M2 polarization, induces CD8^+^ T cell recruitment, and improves sensitivity to radiotherapy (40). CDC25B and CDC25C belong to the CDC25 phosphatase family. Unlike CDC25C, CDC25B promotes G2/M transition by dephosphorylating and activating CDK1 during the G2 phase (10). CDC25B participates in immune pathways similar to CDC25C. CDC25B expression correlates with infiltration of B cells, CD8^+^ T cells, CD4^+^ T cells, Mφ, neutrophils, and dendritic cells in HCC (41).

Immunological features determine the prognosis and therapeutic response in HCC. We found that APIGPS was predominantly expressed in Mφ, with high Mφ abundance in the C1 subtype and HRG. High expression of Mφ aws significantly associated with poor prognosis, and this immune microenvironment remodeling may contribute to immunotherapy resistance, which may also explain the lower abundance of resting memory CD4^+^ T cells in the C1 subtype. Mφ can be categorized into tissue-resident Mφ and monocyte-derived Mφ. During early HCC, tissue-resident Mφ accumulate close to tumor cells, promoting epithelial-mesenchymal transition and tumor invasiveness, while inducing myeloid-derived suppressor cells and Tregs response to form an immunosuppressive microenvironment (42). During tumor progression, tissue-resident Mφ were redistributed at the periphery of the tumor microenvironment, whereas monocyte-derived Mφ dominate the TME. The latter highly express PD-L1, PD-L2, CD80, and CD86, resulting in resistance to radiotherapy, chemotherapy, and immune checkpoint blockade (ICB) therapy (42, 43). Research indicates that Mφ suppress endogenous STAT3 in T cells by releasing CSF - 1, chemokines or exosomes, thereby regulating Treg/Th17 cell balance and continuously recruiting monocyte-derived Mφ into the TME to form a drug-resistant positive feedback loop (44, 45). Additionally, Mφ can capture PD - 1 antibodies through the Fc-FcγR pathway, leading to resistance to ICB, which aligns with our GSVA results that the FcγR pathway was highly expressed in HRG (46). *In vivo* studies showed that Mφ and Tregs are colocalized in tumor tissues. Depletion of Mφ can reverse the immunosuppression in the TME, restore ICB efficacy, and promot CD8^+^ T cell infiltration into HCC cells (47, 48). In addition, Our study revealed that Mφ communicated with endothelial cells via the PPIA/BSG axis. This process activates NF-κB through the IL - 6/STAT3 pathway, upregulates PD-L1 expression, and suppresses T cell activity (49). Targeting the PPIA-CD147 axis can block this cascade, inhibit angiogenesis, and reverse immune escape in HCC. However, it is noteworthy that there are alternative pathways to PPIA/BSG-mediated Mφ-endothelial cell communication, as evidenced by persistent PPIA-induced IL - 8 expression even after BSG knockdown (50). TP53 was the most frequently mutated gene in both HRG and LRG. TP53 mutation frequency was associated with abundance of NK cells, Mφ, and follicular helper T cells in HCC (51). TP53 gain-of-function mutation promote Mφ recruitment via BRD4-dependent CSF - 1 expression (52). Loss of TP53 increases PD-L1 expression and reduces CD8^+^ T cell infiltration in HCC samples and mouse models (53).

GO and KEGG analyses showed that the main differences in Mφ-related pathways between HRG and LRG were leukocyte mediated immunity and chemokine signaling pathway, which suggests Mφ polarization and reprograming of the immune microenvironment. Mφ belong to myeloid-derived leukocytes and exhibit high plasticity: classically activated M1have pro-inflammatory and anti-tumor effects, while alternatively activated M2exert anti-inflammatory and pro-tumor effects. These polarized states can interconvert under specific conditions. Cytokines like IL12, TNF, and IFNG, MAMPs such as LPS, or other Toll-like receptor agonists can induce polarization toward M1 state. Conversely, IL4, IL5, IL10, IL13, CSF1, TFGB1, and PGE2 all promote Mφ polarization toward M2 state. It is worth noting that although targeting M1 polarization is beneficial, the role of M1 polarization appears to be double-edged. M1have been shown to promote HCC cell motility by secreting IL - 1β, and induce PD-L1 expression via IRF1 and p65, thereby contributing to adaptive resistance to immunotherapy in HCC (54). However, this manipulation of Mφ phenotypic plasticity raises several concerns. For instance, can M1acquire M2-associated properties? Will the converted cells exhibit stronger tumor-promoting effects? Moreover, is the simplistic M1/M2 binary classification sufficient to describe macrophage states in the TME? These questions remain to be elucidated through further investigation.

Our study first identified APIG and their potential pathways and mechanisms affecting HCC, constructed a prognostic signature based on them, and further determined the hub gene. However, this study also has several critical limitations that must be acknowledged. The most significant limitation is the lack of comprehensive experimental validation. Although we performed qRT-PCR to confirm the expression levels of target genes, this alone is insufficient to establish causal relationships or mechanistic insights. *In vitro* functional experiments, such as siRNA-mediated gene knockdown, CCK - 8 assays to evaluate cell proliferation, flow cytometry or immunofluorescence to assess Mφ polarization, and Transwell migration assays are essential to verify the functional roles of the identified APIGs. Moreover, *in vivo* studies would provide important mechanistic and translational validation. The absence of these validations may currently weaken the biological conclusions of the study. Second, the specificity of the effect of APs on HCC remains largely unclear. Clinically, multiple APs often coexist and may interact synergistically (e.g., ozone, benzene/toluene), making it challenging to determine the individual contribution of each pollutant. Further studies are needed to clarify the pollutant-specific mechanisms driving HCC. Third, the affinity between APs and Mφ and the polarization direction (M1/M2) remain unclear. More importantly, it is unknown whether AP exposure can actively trigger Mφ repolarization, and how the temporal dynamics of such polarization evolve. Single-cell and transcriptomics analyses are recommended to dissect spatiotemporal changes in the immune microenvironment. Furthermore, although molecular docking analyses suggested potential binding between APs and target proteins, these predictions do not confirm causal regulatory relationships. Future studies should incorporate *in vitro* or *in vivo* AP exposure experiments to examine the dynamic regulation of APIG expression, and perform transcriptomic profiling under controlled exposure conditions to elucidate regulatory mechanisms. In addition, while the APIGPS demonstrates robust performance in the current datasets, there remains a risk of overfitting due to reliance on retrospective public cohorts. The generalizability of the model to broader clinical populations or real-world settings is yet to be established. Prospective validation in large, multi-center cohorts is necessary to assess its clinical applicability. Finally, while this study suggests APs may impact immunotherapy efficacy in HCC, and green environment or pollution levels correlate with patient prognosis, the mechanistic links between APs and immunotherapy warrants further exploration.

## Conclusions

Our study identified 19 APIGs from 7 APs, and selected 7 of them to construct an APIGPS with good predictive performance. Among them, CDC25C was the hub gene with diagnostic and prognostic value, and was associated with the survival outcomes in 10 cancers. Future studies should include experimental validation using AP extracts, employing techniques such as siRNA, CCK - 8, flow cytometry, immunofluorescence, Western blotting, and Transwell assays to further elucidate the molecular mechanisms and immune regulatory effects of APIGs. Overall, this study underscores the importance of integrating environmental exposure factors into cancer research, particularly in understanding HCC progression and improving individualized prognosis and treatment strategies.

## Data Availability

The original contributions presented in the study are included in the article/**Supplementary Material**. Further inquiries can be directed to the corresponding author.
