# Supplementary material for: Air pollution-related immune gene prognostic signature for hepatocellular carcinoma: network toxicology, machine learning and multi-omics analysis
Source: Front Immunol. 2025 Sep 12;16:1638445. doi: 10.3389/fimmu.2025.1638445 (PMC12463942; doi:10.3389/fimmu.2025.1638445)
Supplement: Supplementary file 1 [file DataSheet1.pdf]

Air pollution-related immune gene prognostic signature for hepatocellular carcinoma: network toxicology, machine learning and multi-omics analysis

Lei Pu[1, #], Xiaoyan Zhang[1, #], Cheng Pu[2], Peng Sun[1, *]

[1] Key Laboratory of Adolescent Health Assessment and Exercise Intervention of the Ministry of Education, East China Normal University, Shanghai 200241, P.R. China

[2] College of Martial Arts, Shanghai University of Sport, Shanghai 200438, P.R. China

*** Corresponding author**

Peng Sun, Ph.D.

Key Laboratory of Adolescent Health Assessment and Exercise Intervention of the Ministry of Education, East China Normal University, 500 Dongchuan Road, Shanghai 200241, China.

Email: psun@tyxx.ecnu.edu.cn

# Co-first authors

Lei Pu and Xiaoyan Zhang are co-first authors.

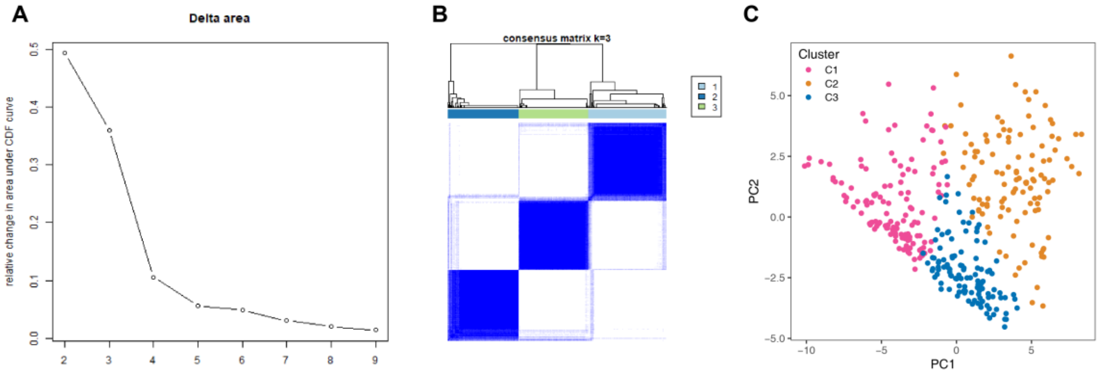

Fig. S1 Molecular subtyping. (A) Relative change in area under CDF curve. (B) three subtypes of HCC. (C) PCA of the three subtypes.

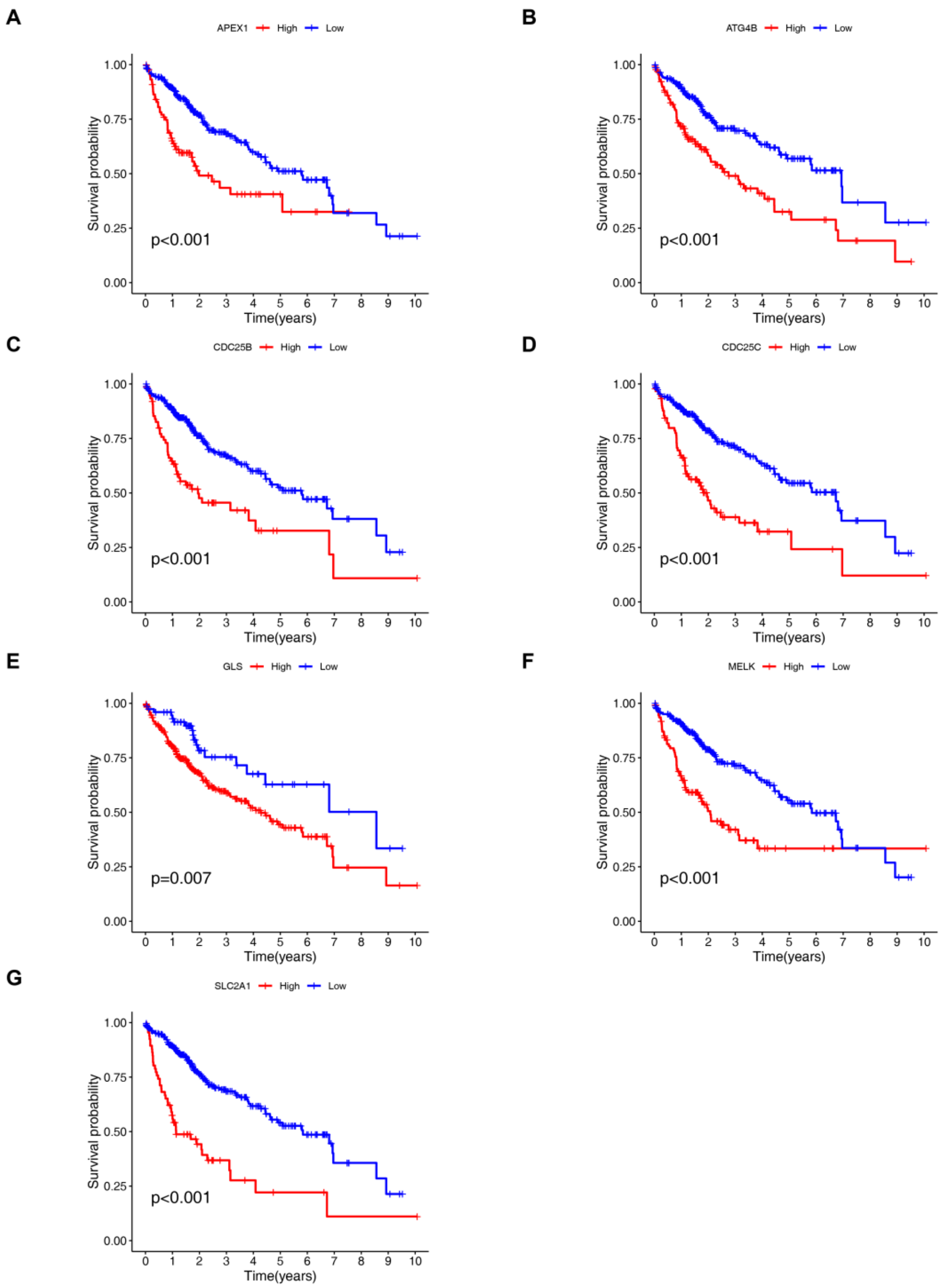

Fig. S2 Survival analysis of APIGPS genes. (A-G) Survival analysis of 7 APIG genes.

Fig. S3 Immunohistochemical analysis obtained by HPA. (A-G) All 7APIG are highly expressed in HCC.

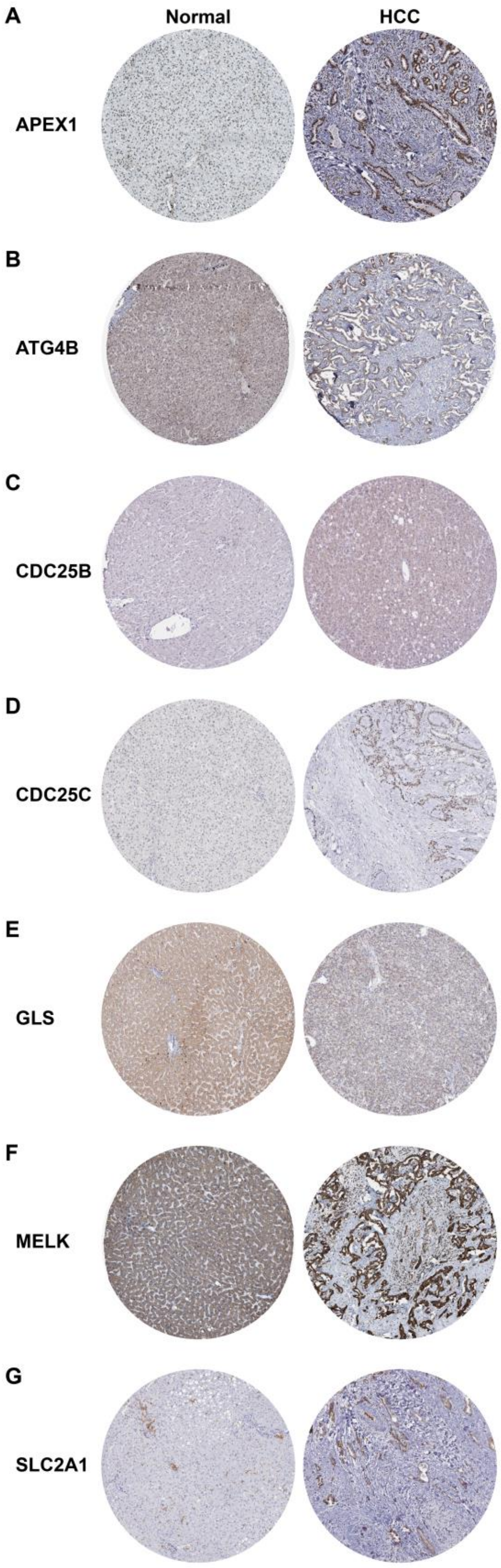

Fig. S3 Immunohistochemical analysis obtained by HPA. (A-G) All 7APIG are highly expressed in HCC.

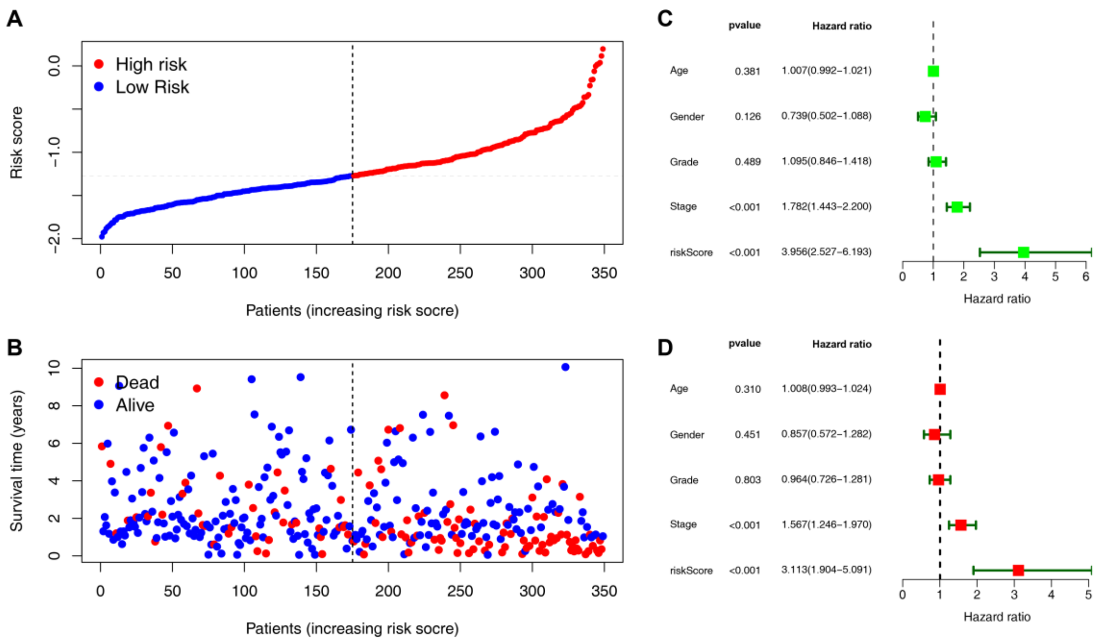

Fig. S4 Performance evaluation of APIGPS. (A) Risk curves based on APIGPS. (B) Scatter plot of risk scores. (C) Univariate COX regression analysis of risk scores. (D) Multivariate COX regression analysis of risk scores.

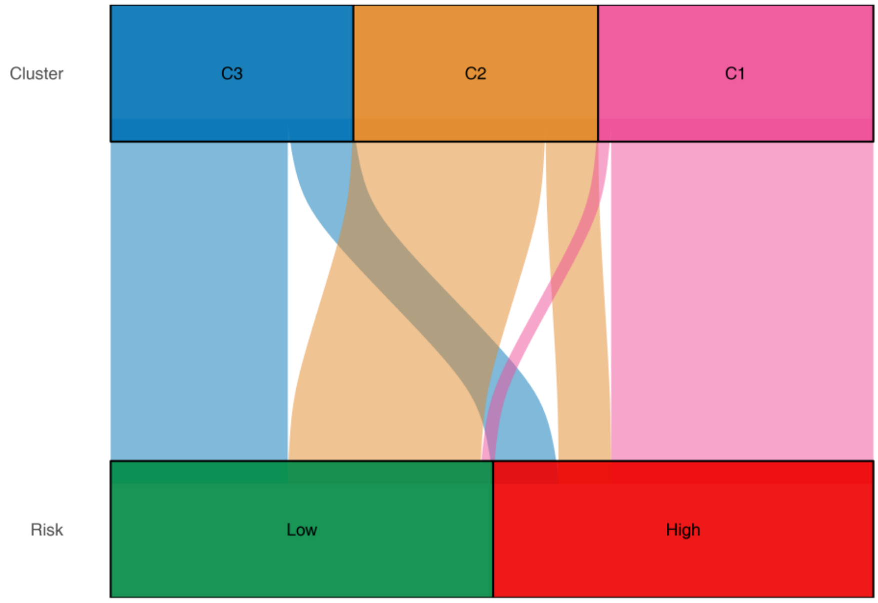

Fig. S5 The association between risk groups and molecular subtypes.

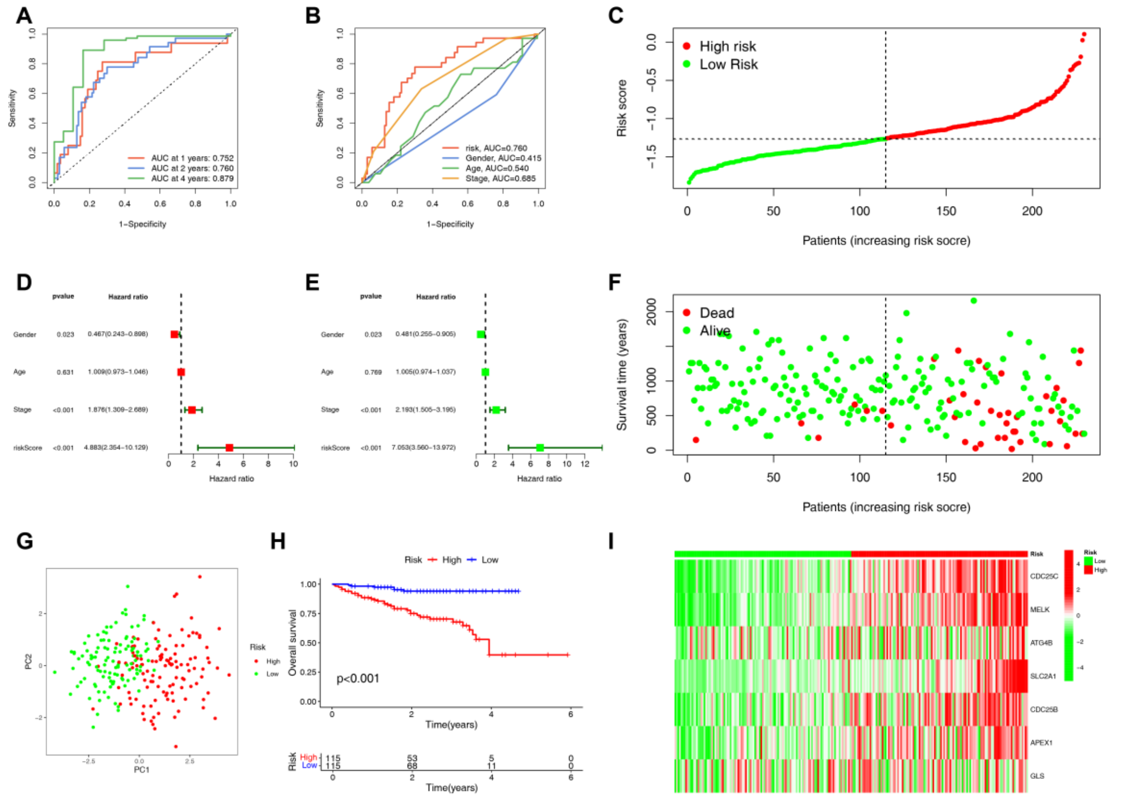

Fig. S6 External validation of EIGPS. (A) ROC curves predicting 1-, 2-, and 4-year survival. (B) ROC curves for clinical features and risk scores. (C) Risk score curves. (D) Multivariate COX regression analysis of risk scores. (E) Univariate COX regression analysis of risk scores. (F) Scatter plot of risk scores. (G) PCA based on risk scores. (H) K-M survival analysis. (I) Heat map of signature gene expression.

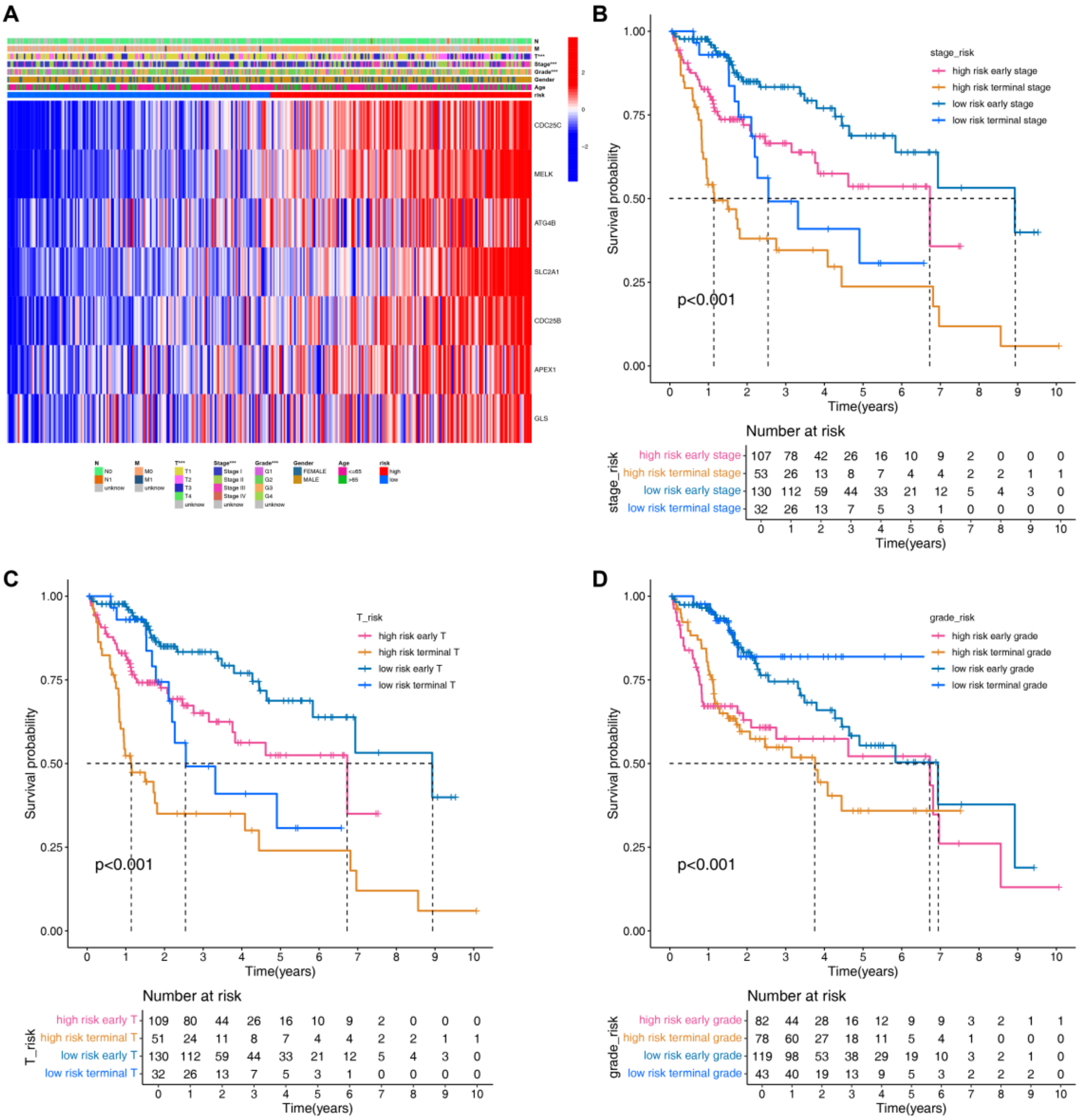

Fig. S7 Construction of clinical features and nomogram. (A) Heatmap of clinical features and signature gene expression in the APIGPS. (B) Survival analysis combining risk scores and stage. (C) Survival analysis combining risk scores and T-stage. (D) Survival analysis combining risk scores and grade.

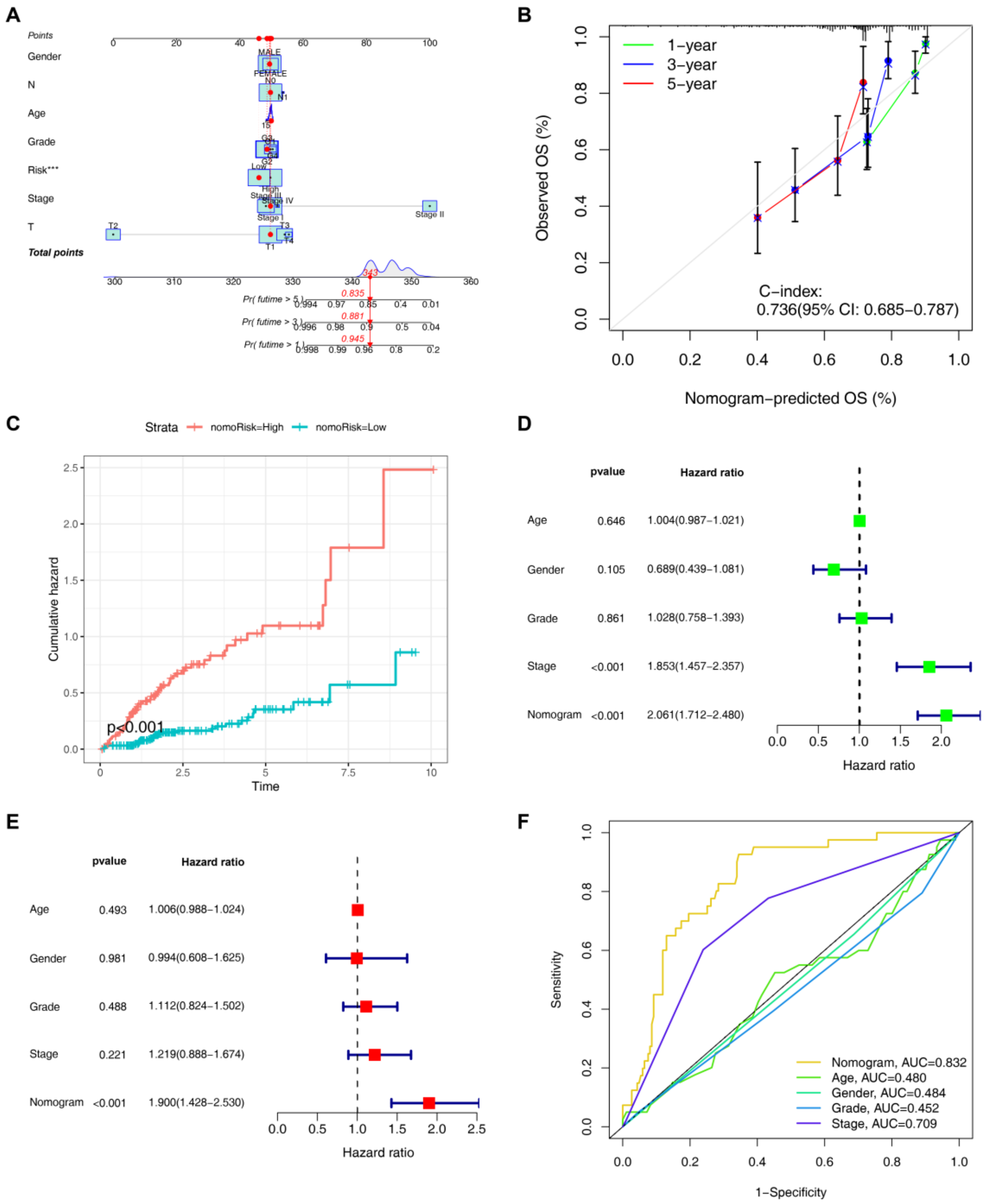

Fig. S8 Construction and validation of the nomogram. (A) The nomogram based on clinical features and risk scores. (B) C-index values of the nomogram based on clinical features and risk scores. (C) Survival analysis of nomogram scores. (D) Univariate COX analysis of nomogram. (E) Multivariate COX analysis of nomogram. (F) ROC curves for clinical features and nomogram.

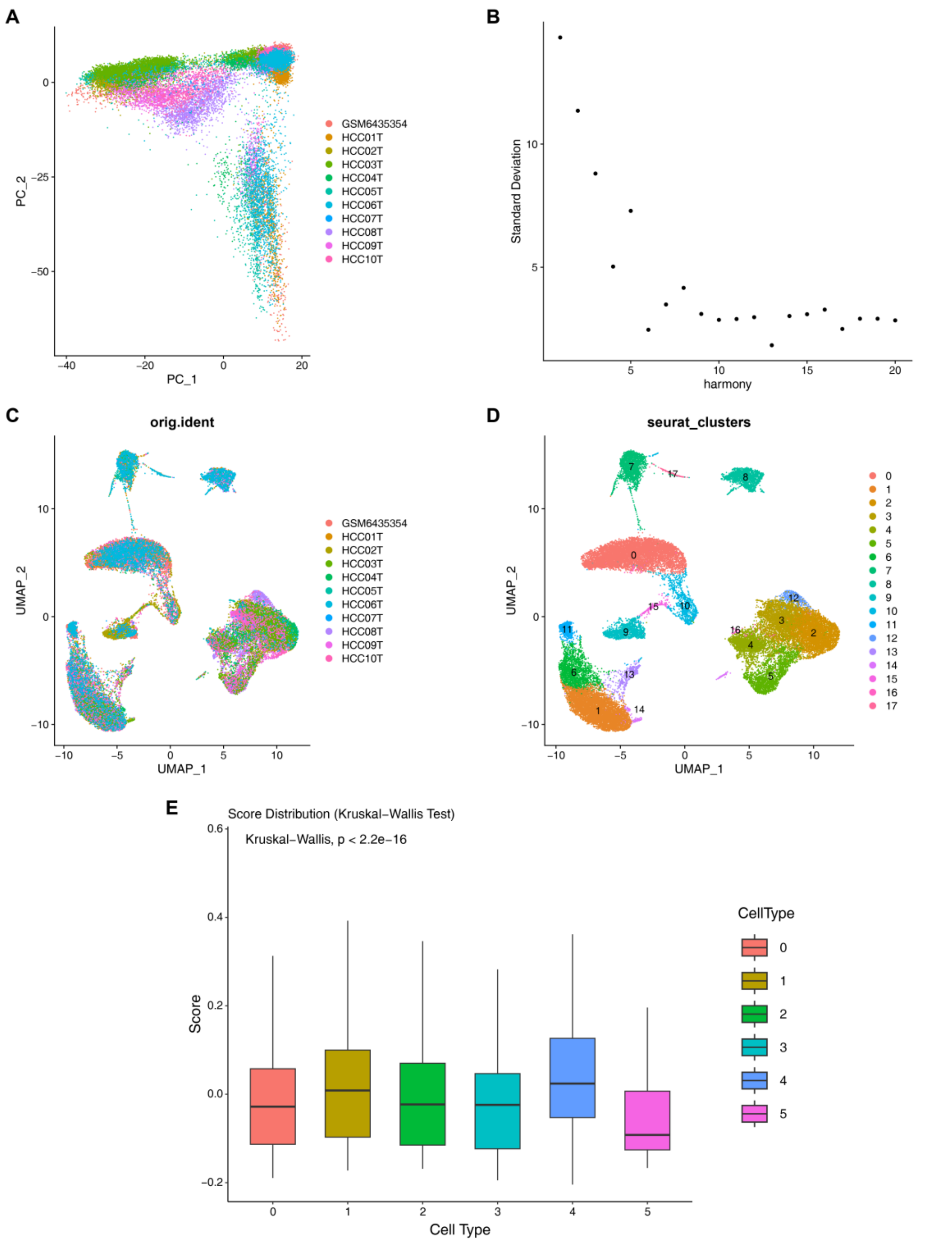

Fig. S9 ScRNA analysis of HCC. (A) PCA. (B) Elbow plot. (C) Clustering analysis of samples. (D) 18 clusters with 0.5 as the best resolution. (E) APIGPS of score in 6 clusters

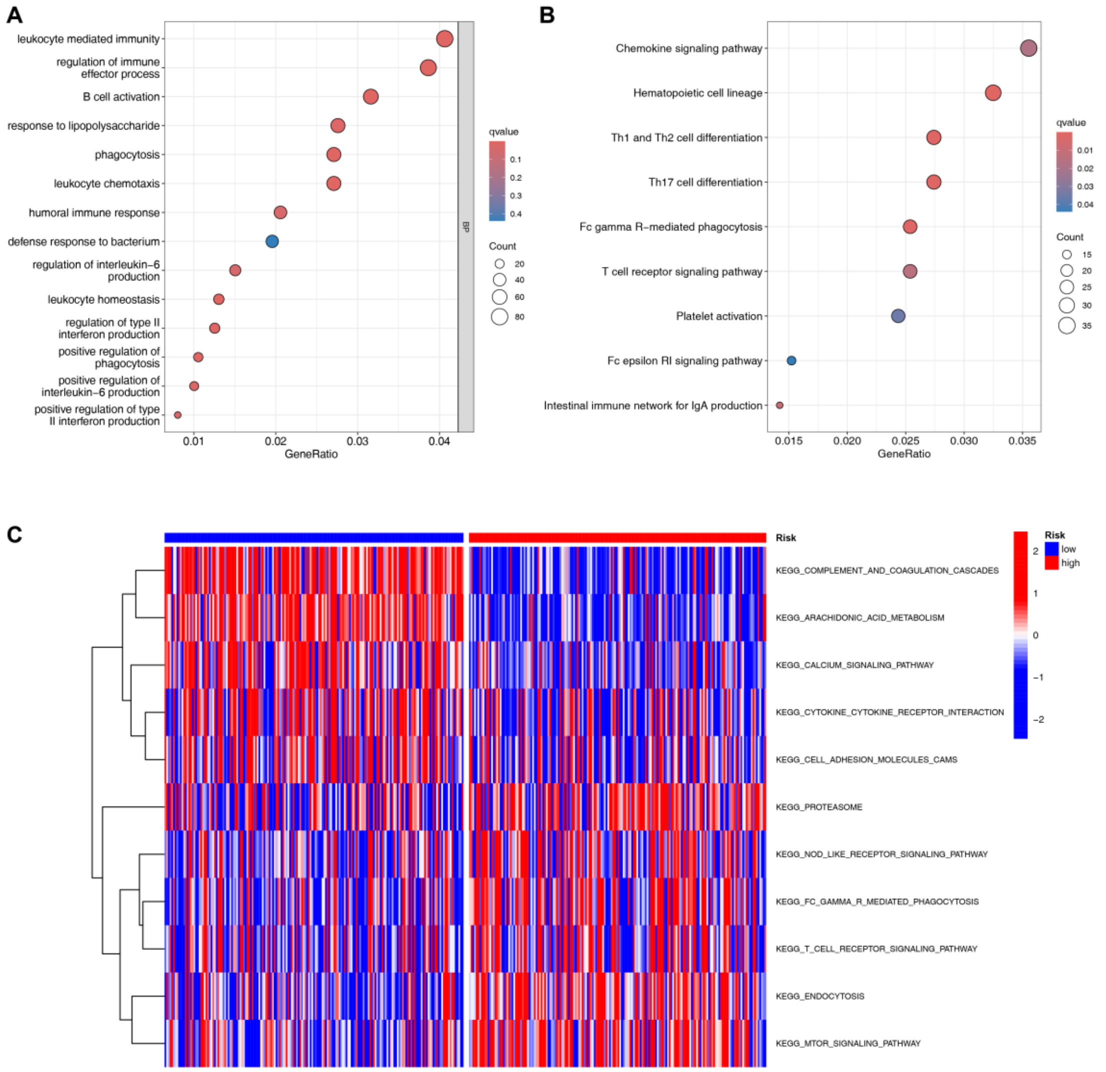

Fig. S10 Macrophage-related immune pathway analysis. (A) GO analysis. (B) KEGG analysis. (C) GSVA analysis.

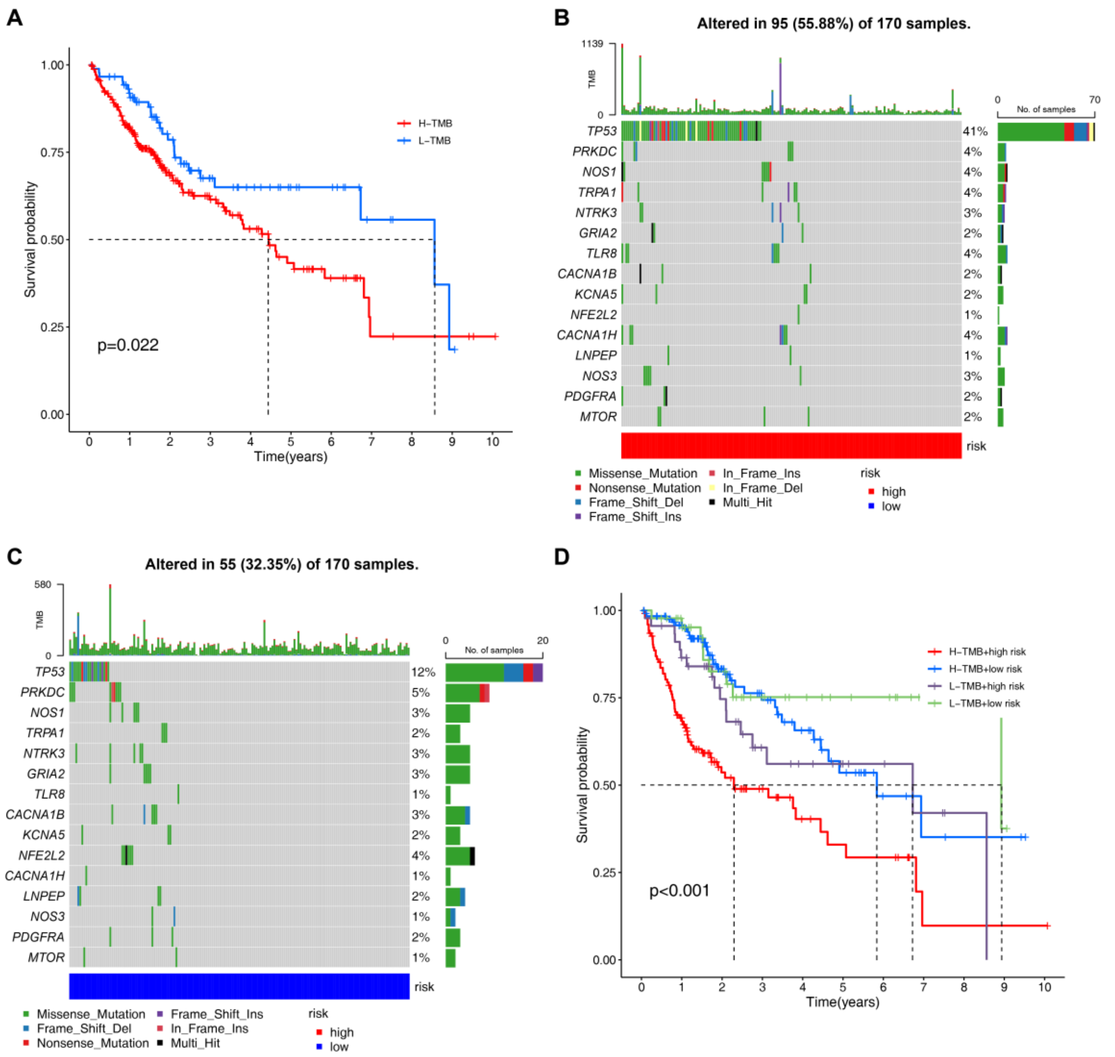

Fig. S11 Tumor mutation burden analysis. (A) Survival analysis of high and low TMB groups. (B) Mutation frequency in the HRG. (C) Mutation frequency in the LRG. (D) Survival analysis combining risk scores and high and low TMB groups.

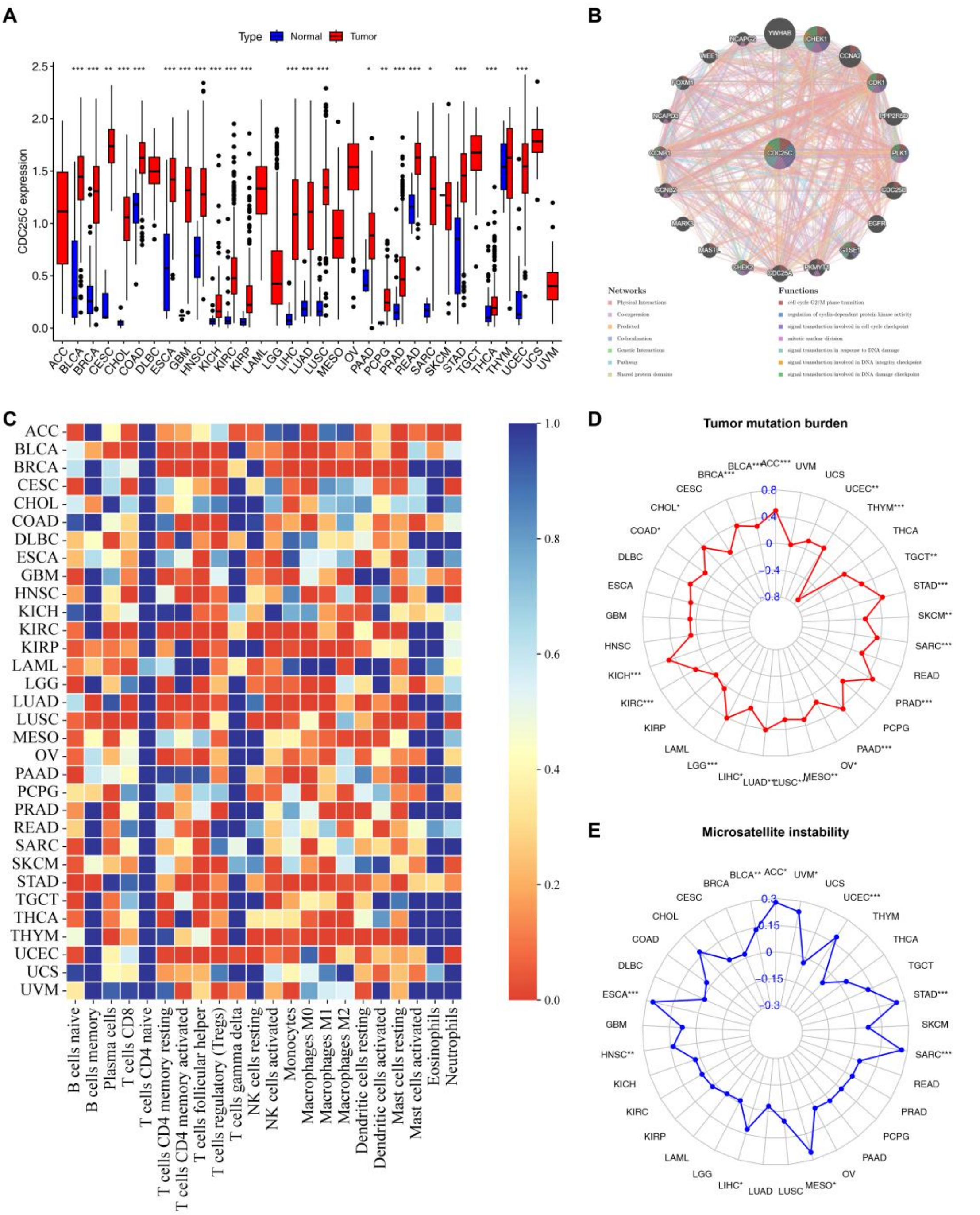

Fig. S12 Pan-cancer analysis of hub gene. (A) Expression of CDC25C across cancers. (B) Functions of CDC25C. (C) Correlation of CDC25C with immune cell infiltration across cancers. (D) Correlation of CDC25C with tumor mutation burden across cancers. (E) Correlation of CDC25C with MSI across cancers.

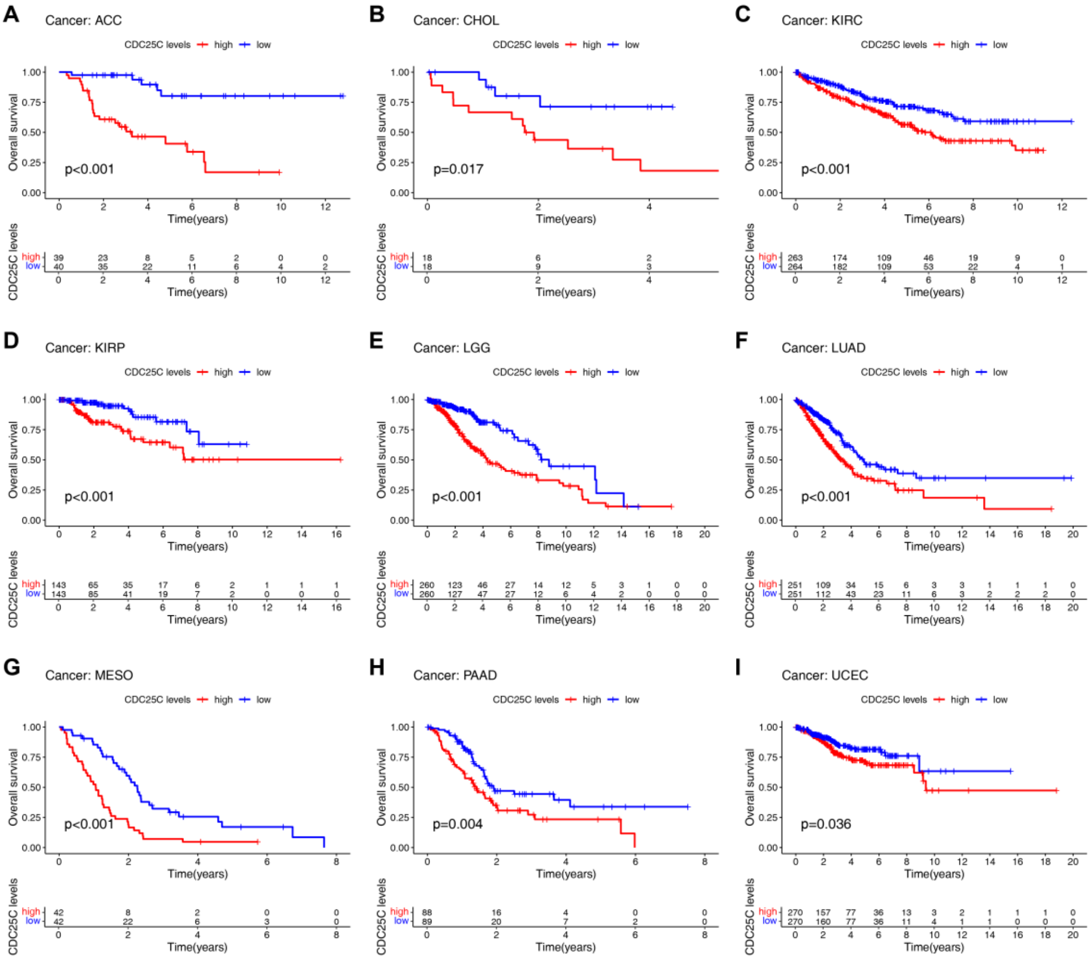

Fig. S13 High expression of CDC25C is associated with poor survival in 9 types of cancer other than HCC. (A) Adrenocortical carcinoma. (B) Cholangiocarcinoma. (C) Kidney renal clear cell carcinoma. (D) Kidney renal papillary cell carcinoma. (E) Brain Lower Grade Glioma. (F) Lung adenocarcinoma. (G) Mesothelioma. (H) Pancreatic adenocarcinoma. (I) Uterine Corpus Endometrial Carcinoma.
